# Immune microenvironment characterisation and dynamics during anti-HER2-based neoadjuvant treatment in HER2-positive breast cancer

**DOI:** 10.1038/s41698-021-00163-6

**Published:** 2021-03-19

**Authors:** G. Griguolo, G. Serna, T. Pascual, R. Fasani, X. Guardia, N. Chic, L. Paré, S. Pernas, M. Muñoz, M. Oliveira, M. Vidal, A. Llombart-Cussac, J. Cortés, P. Galván, B. Bermejo, N. Martínez, R. López, S. Morales, I. Garau, L. Manso, J. Alarcón, E. Martínez, P. Villagrasa, A. Prat, P. Nuciforo

**Affiliations:** 1 Department of Surgery, Oncology and Gastroenterology, University of Padova, Padova, Italy; 2 Division of Oncology 2, Istituto Oncologico Veneto IRCCS, Padova, Italy; 3 Translational Genomics and Targeted Therapeutics in Solid Tumors, August Pi i Sunyer Biomedical Research Institute (IDIBAPS), Barcelona, Spain; 4 Molecular oncology group, Vall d´Hebron Institute of Oncology, Barcelona, Spain; 5 Department of Medical Oncology, Hospital Clínic de Barcelona, Barcelona, Spain; 6 SOLTI Breast Cancer Research Group, Barcelona, Spain; 7 Institut Catala d’Oncologia-H.U.Bellvitge-IDIBELL, Hospitalet, Barcelona, Spain; 8 Medical Oncology Department, Vall d’Hebrón University Hospital, Barcelona, Spain; 9 Breast Cancer and Melanoma Group, Vall d’Hebron Institute of Oncology, Barcelona, Spain; 10 Hospital Universitario Arnau de Vilanova de Valencia, Valencia, Spain; 11 IOB Institute of Oncology, Quironsalud Group, Madrid & Barcelona, Spain; 12 Hospital Clínico Universitario de Valencia/INCLIVA/CIBERONC, Valencia, Spain; 13 Hospital Universitario Ramón y Cajal, Madrid, Spain; 14 Hospital Clínico Universitario de Santiago, IDIS, CIBERONC, Santiago de Compostela, Spain; 15 Hospital Universitario Arnau de Vilanova de Lleida, Lleida, Spain; 16 Hospital Son Llàtzer, Palma de Mallorca, Spain; 17 Hospital Universitario 12 de Octubre, Madrid, Spain; 18 Hospital Universitario Son Espases, Palma de Mallorca, Spain; 19 Consorcio Hospitalario Provincial de Castellón, Castellón de la Plana, Spain

**Keywords:** Breast cancer, Tumour immunology, Tumour heterogeneity, Breast cancer

## Abstract

Despite their recognised role in HER2-positive (HER2+) breast cancer (BC), the composition, localisation and functional orientation of immune cells within tumour microenvironment, as well as its dynamics during anti-HER2 treatment, is largely unknown. We here investigate changes in tumour-immune contexture, as assessed by stromal tumour-infiltrating lymphocytes (sTILs) and by multiplexed spatial cellular phenotyping, during treatment with lapatinib-trastuzumab in HER2+ BC patients (PAMELA trial). Moreover, we evaluate the relationship of tumour-immune contexture with hormone receptor status, intrinsic subtype and immune-related gene expression. sTIL levels increase after 2 weeks of HER2 blockade in HR-negative disease and HER2-enriched subtype. This is linked to a concomitant increase in cell density of all four immune subpopulations (CD3^+^, CD4^+^, CD8^+^, Foxp3^+^). Moreover, immune contexture analysis showed that immune cells spatially interacting with tumour cells have the strongest association with response to anti-HER2 treatment. Subsequently, sTILs consistently decrease at the surgery in patients achieving pathologic complete response, whereas most residual tumours at surgery remain inflamed, possibly reflecting a progressive loss of function of T cells. Understanding the features of the resulting tumour immunosuppressive microenvironment has crucial implications for the design of new strategies to de-escalate or escalate systemic therapy in early-stage HER2+ BC.

## Introduction

The host immune system has an important role in HER2-positive (HER2+) breast cancer (BC). Prior studies have revealed that ~55% of HER2+ tumours have >10% of stromal tumour-infiltrating lymphocytes (sTILs)^1^. From a clinical point of view, TILs are associated with better survival outcomes in HER2+ early and advanced BC^1–3^, higher pathological complete response (pCR) rates after neoadjuvant anti-HER2-based chemotherapy^2,4–7^ and higher response to trastuzumab plus pembrolizumab in the advanced setting^8^. Thus, baseline TILs in HER2+ disease determine prognosis and might contribute to the therapeutic effects of anti-HER2-based treatments^9,10^.

Despite the recognised role of immune cells in HER2+ BC, the composition, localisation and functional orientation of immune cells within the tumour microenvironment (jointly referred to as immune contexture), as well as the dynamics of TILs during and after anti-HER2 treatment, are largely unknown. Limited and inconsistent evidence is available regarding the prognostic impact of TILs in residual tumours following neoadjuvant anti-HER2-based chemotherapy. High TIL levels in residual disease have been associated both with better outcome^11^, worse outcome^12^ and no impact on prognosis^13^. A study by Ladoire S. and colleagues evaluated the prognostic impact of different lymphocytic subpopulations; it reported high CD8 and low Foxp3 cell infiltrates after chemotherapy to be significantly associated with improved long-term outcome^14^. This study thus further highlights the need for a more comprehensive evaluation.

The current treatment standard of early-stage HER2+ BC is anti-HER2-therapy plus chemotherapy. Thus, prior studies have not been able to dissect whether the observed changes in the immune microenvironment are owing to chemotherapy, anti-HER2 therapy, or both. Studies without chemotherapy are the ideal scenario to address the specific role of anti-HER2 therapy^15,16^. The neoadjuvant PAMELA trial (SOLTI-1114)^15^ treated 151 patients with HER2+ BC with trastuzumab and lapatinib (and endocrine therapy if the tumour was hormone receptor [HR] positive) for 18 weeks. In this study, sTILs at baseline and at day 15 differed significantly according to PAM50 intrinsic subtype. Moreover, sTILs at baseline were found significantly associated with pCR in the univariable analysis but not in multivariable analysis. At day 15, a significant increase in sTILs was observed in most patients; also, sTILs at day 15 were found independently associated with pCR at multivariate analysis^17^.

With this background, several questions can be addressed from the PAMELA trial: (1) which cells compose immune infiltrate in early HER2+ BC and how are they interacting with tumour cells?; (2) how does this relate to the probability of achieving a pCR?; (3) which subgroup of patients increases sTILs after 2 weeks of priming with HER2-targeted treatment?; (4) how do these changes relate to the probability of achieving a pCR?; (5) how do immune contexture changes after anti-HER2 priming relate to the probability of achieving a pCR?; (6) how are sTILs expressed at surgery following neoadjuvant treatment? and (7) how is the presence of sTILs associated with immune-related gene expression?

We here investigated changes in the tumour-immune microenvironment following treatment with lapatinib and trastuzumab and the relationship of sTILs with HR status, intrinsic subtype and immune-related gene expression in patients with HER2+ BC from the PAMELA trial. Moreover, we assessed immune contexture at baseline and day 15 in patients with available samples by multiplexed spatial cellular phenotyping (REMARK diagram, Supplementary Fig. 1). The results from this analysis might help design new strategies to de-escalate or escalate systemic therapy in HER2+ early BC.

## Results

### A multiplexed imaging assay for immune microenvironment characterisation

**Fig. 1:**
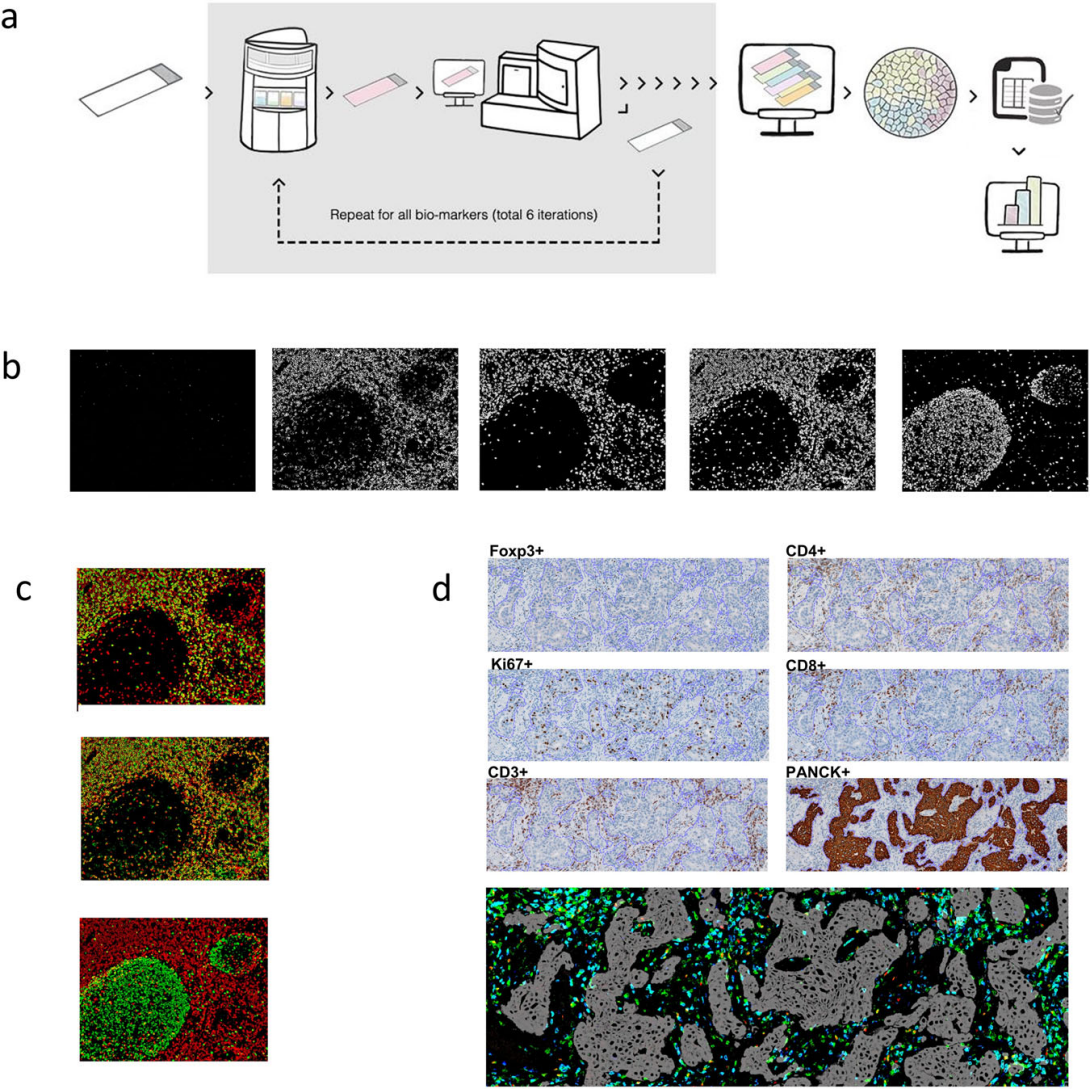
Multiplexed imaging assay. **a** Next-generation immunohistochemistry (NGI) workflow. An FFPE tissue section is stained, scanned and destained six times. All the scanned images are aligned, image analysis is done to obtain the data and after doing all the quality check controls, the data are analysed to obtain the final results. **b** Representative colour deconvoluted images of different biomarkers in the tonsil. From left to right Foxp3^+^, CD3^+^, CD8^+^, CD4^+^ and Ki67^+^. Images at 6×. **c** Colour overlays of different biomarkers in the tonsil (CD3^+^ in red, CD8^+^, CD4^+^ and Ki67^+^ in green from top to bottom). Images at 6×. **d** A representative example of all the stainings (Foxp3, CD4, KI67, CD8, CD3 and cytokeratin) in breast cancer samples and virtual image reconstruction of some of them by assigning virtual colours to the deconvoluted images. Foxp3^+^ in red, CD8^+^ in blue, CD3^+^ in green and cytokeratin in grey. The fine purple line in each image marks the tumour borders. Images at 5×.

We developed a multiplexed immunohistochemistry (IHC) workflow (named next-generation IHC or next-generation impactor (NGI)) comparable with that of conventional IHC. This workflow is based upon iterative cycles of staining and destaining of the same slide with different primary antibodies, individual slide digitalisation, virtual multiplexed digital image reconstruction and complex image analyses (Fig. 1a). A six-plex panel was specifically designed to interrogate the tumour-immune microenvironment and included a tumour-related protein (cytokeratin), a functional marker for proliferation (Ki67) and four immune-related T-cell lineage markers (CD3, CD4, CD8 and Foxp3). The latter was selected as established markers of T cells with an effector (CD3^+^ CD8^+^) and suppressor/regulatory (CD3^+^CD4^+^Foxp3^+^) functions.

The antibody staining of each target was initially optimised using two different tissue controls (a normal tonsil and a BC) and further validated on an independent series of HER2+ BC stained by regular IHC to ensure consistent results among methods (Supplementary Figs. 2 and 3). We evaluated marker specificity by matching the staining obtained with each antibody to the known histologic distribution and to staining with a conventional IHC protocol: Ki67-stained cells in the germinal centre, and CD3-, CD4- and CD8-stained cells predominantly in the mantle zone (Fig. 1b, c). Furthermore, we evaluated the subcellular localisation of the biomarkers and confirmed that staining of the transcription factors Ki67 and Foxp3 were nuclear, whereas CD3, CD4 and CD8 were membrane.

The individual digitalised IHC images were aligned to obtain a virtual multiplexed image (Fig. 1d), which was analysed using different image analysis algorithms developed for biomarker densities, function and spatial analyses (Supplementary Fig. 4).

### Immune microenvironment contexture

A total of 231 regions of interest (ROI) from 129 unique samples had sufficient material for NGI analysis. All samples were stained with a sequential IHC workflow that included a panel of six antibodies for T cells subtyping (CD3, CD4, CD8 and Foxp3), proliferation (Ki67) and tumour recognition (cytokeratin) plus hematoxylin for counterstaining. Samples with total region of interest (ROI) below 100,000 µm were excluded from the analysis. After filter, a total of 114 samples (65 baseline and 49 day 15) from 75 patients were evaluable. An average (range) ROI of 7,764,692 µm^2^ (122,074–37,742,587) was profiled using an image analysis pipeline for data extraction and analysis (see methods).

**Fig. 2:**
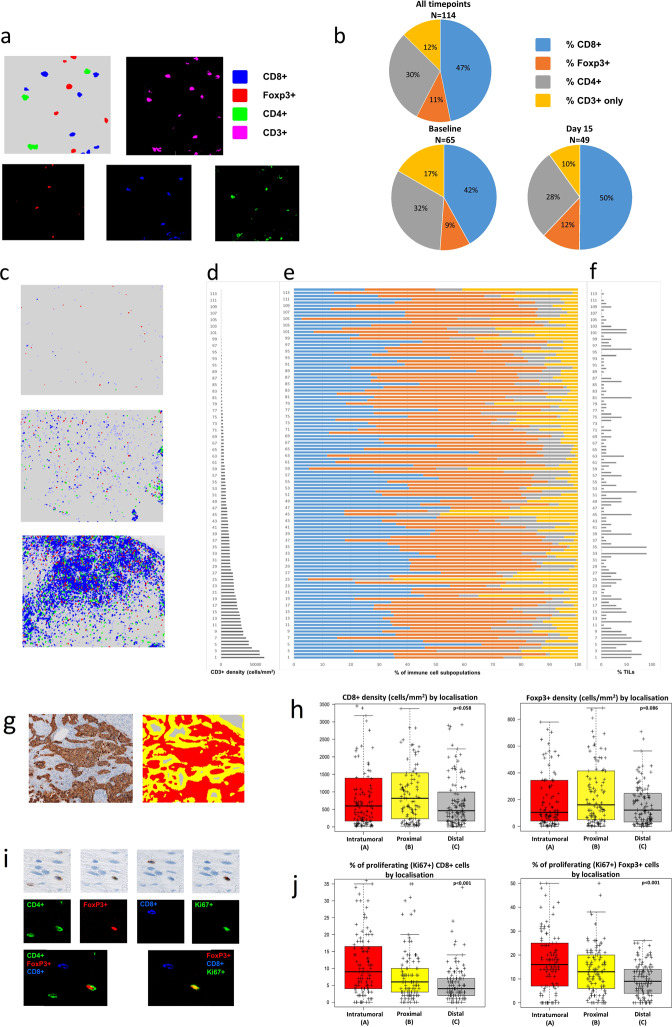
Multiplexed spatial cellular phenotyping of breast cancer. **a** Representative example of the analytical pipeline classifying immune cells (top left) and colour deconvoluted images with red, green and blue colours assigned to Foxp3^+^, CD4^+^ and CD8^+^, respectively (bottom) and magenta for CD3^+^ (top right). Images at 25×. **b** Proportions of CD3^+^ immune subsets across all patients’ samples (up), baseline samples (down to the left) and day 15 samples (down to the right). CD8^+^, Foxp3^+^, CD4^+^ and CD3^+^-only in blue, orange, gray and yellow. **c** Representative examples of breast cancers with low (up), medium (middle) and high (down) T-cell densities. Images at 5×. **d** CD3^+^ density results across the entire population of HER2+ breast cancers. Samples are ordered from lowest to highest. **e** Proportions of CD8^+^ (blue), Foxp3^+^ (orange), CD4^+^ (gray) and CD3^+^ only (yellow) cells for all patients’ samples. **f** Stromal tumour-infiltrating lymphocytes (TILs) in breast cancer samples with available NGI data. **g** Representative example of the spatial analysis areas defined by the image analysis algorithm using cytokeratin as tumour mask. Intratumoural (**a**, in red), proximal peritumoural stroma within 30 µm (**b**, in yellow) and distal peritumoural stroma >30 µm from the tumour (**c**, in gray) regions are shown. Images at 5×. **h** Boxplots of immune cells densities (CD8^+^ and Foxp3^+^) according to spatial location. Boxplot legend: centre line: median; bounds of box: interquartile range (IQR); whiskers: highest and lowest value excluding outliers (Q3 + 1.5*IQR to Q1 − 1.5*IQR); markers beyond the whiskers: potential outliers. **i** Representative example of CD4^+^, Foxp3^+^, CD8^+^ and KI67^+^ sequential immunohistochemistry and co-expression analyses for T cells activity assessment on the same tissue slide (top panel). The colour green, red, blue and green is assigned, respectively, to each individual staining for visualisation purpose, co-expression analyses and virtually multiplexed images (first image composed by CD4, Foxp3 and CD8 and second image composed by Foxp3, CD8 and Ki67; co-expression in yellow). Images at 50×. **j** Boxplots of the proportion of proliferating immune cells (CD8^+^ and Foxp3^+^) according to spatial location. Boxplot legend: centre line: median; bounds of box: interquartile range (IQR); whiskers: highest and lowest value excluding outliers (Q3 + 1.5*IQR to Q1 − 1.5*IQR); markers beyond the whiskers: potential outliers.

To identify the immune cell subpopulations within the samples, we clustered the immune cells by canonical markers. We found that our analytical pipeline was able to accurately classify immune cells even when those with opposite identifiers were located in close proximity to each other (Fig. 2a). Over a total of 1,217,249 cells identified, the proportions of CD3^+^ immune subsets across all patients’ samples were 47% CD8^+^CD4^−^Foxp3^−^, 30% CD4^+^Foxp3^−^CD8^−^, 11% CD4^+^Foxp3^+^CD8^−^ and 13% CD8^−^CD4^−^Foxp3^−^ (Fig. 2b).

To chart the immune landscape in HER2+ BC, we then quantified the number of immune cell populations per area across patients. We found large variability in immune cells content, which was confirmed by pathological sTILs scoring on H&E staining (Supplementary Table 1, Fig. 2c–f, Supplementary Figs. 5–6). Densities of all immune cell subtypes were all positively correlated with the number of sTILs (Spearman Rho, CD3^+^ = 0.63, CD8^+^ = 0.65, CD4^+^ = 0.54, Foxp3^+^ = 0.615, *P* < .0001, Supplementary Fig. 7) assessed by a board-certified pathologist according to international TILs working group recommendation^18^. Densities of all immune cell subtypes negatively correlated with the percentage of tumour area in the sample (CD3^+^ = −0.209, *P* = 0.028; CD8^+^ = −0.205, *P* = 0.031; CD4^+^ = −0.184, *P* = 0.053, Foxp3^+^ = −0.203, *P* = 0.033, Supplementary Fig. 8a).

To evaluate the spatial organisation of the tumour-immune landscape in HER2+ BC, we developed a method for assessing spatial proximity enrichment of each immune cell subtype from the tumour. We quantified the number of positive cells for each marker located within three regions of different distance from the tumour: A, intratumoural; B, proximal stroma within 30 µm and C, distal stroma >30 µm from the tumour, respectively (Fig. 2g).

Higher immune cells densities were found in the proximal peritumoural regions (B) compared with intratumoural (A) and distal peritumoural (C) locations although the difference was statistically significant only between location B and C for all immune subtypes (*P* values for comparison between immune cell density in area B and C, CD3 = 0.017, CD8 = 0.014, CD4 = 0.045, Foxp3 = 0.023, Fig. 2h, Supplementary Fig. 9).

To determine the level of activation of immune cells within the tumour microenvironment, we developed a method to quantify the proportion of proliferating immune cells within the stromal compartment by virtually multiplexing individual immune cell markers with Ki67 staining obtained from the same slide (Fig. 2i). The proportion of proliferating immune cells over total proliferating cells (Ki67^+^ tumour and immune cells) was 35%, with tumour cells representing the main proliferative cell subtype within the tissue, as expected (Supplementary Fig. 10). The proportion of proliferating stromal immune cells was positively correlated with the amount of tumour area in the sample (Spearman Rho, CD3^+^Ki67^+^ = 0.397, CD8^+^Ki67^+^ = 0.331, CD4^+^Ki67^+^ = 0.414, Foxp3^+^Ki67^+^ = 0.395, *P* < 0.001, Supplementary Fig. 11).

Spatial analysis revealed a differential distribution according to immune cells proximity to the tumour, with a decreasing proportion of proliferating CD3^+^ (*P* < 0.001), CD8^+^ (*P* < 0.001), CD4^+^ (*P* < 0.001) and Foxp3^+^ (*P* < 0.001) T cells from the intratumoural region (A) to the distal location (C) (Fig. 2j, Supplementary Fig. 12, Supplementary Table 2).

### Immune contexture analysis according to HR status

To evaluate if the tumour-immune microenvironment differed according to HR status, we compared the composition, spatial distribution and functional activity of immune subtypes in HR+ and HR− HER2+ BC.

In the whole sample area, densities of all immune cell subtypes were significantly higher in HR− as compared with HR+ tumours [HR− median (interquartile range) CD3^+^ 1669(1818), CD8^+^ 689(955), CD4^+^ 629(884), Foxp3^+^ 187(215); HR+ median (interquartile range) CD3^+^ 828(863), CD8^+^ 383(520), CD4^+^ 361(472), Foxp3^+^ 57(89); *P* values, CD3^+^ = 0.009, CD8^+^ = 0.009, CD4^+^ = 0.019 and Foxp3^+^ < 0.001, Supplementary Fig. 13a]. As expected, these findings paralleled what observed for sTILs both at baseline and day 15, for which however the difference between HR− and HR+ tumours was only significant after 2 weeks of anti-HER2 treatment (median sTILs at day 15 20% vs 10% in HR− and HR+ tumours, respectively, *P* < 0.001).

The number of immune cells was higher in HR− compared with HR+ across all peritumoural stroma locations, whereas their intratumoural content did not differ significantly according to HR status (Supplementary Fig. 14, Supplementary Table 3).

We further investigated if immune cells activation was influenced by tumour HR status. The proportion of proliferating immune cells was higher in HR- as compared with HR+ tumours (Wilcoxon, CD3^+^ *P* = 0.007; CD8^+^ *P* = 0.011; CD4^+^ *P* = 0.009) except for Foxp3^+^ (*P* = 0.710) (Supplementary Fig. 13b, Supplementary Table 4). These differences were maintained across all peritumoural stroma locations (Supplementary Fig. 15). In HR+, a higher mean proportion of Foxp3^+^ proliferating cells (13.2%) was observed compared with proliferating CD3^+^ (5.6%), CD4^+^ (6.2%) and CD8^+^ (5.4%) (Kruskal–Wallis test, *P* < 0.001). The ratio of proliferating Foxp3^+^/CD8^+^ was significantly higher in HR+ as compared to HR− tumours (2.29 vs 1.33, *P* = 0.004).

### Immune contexture analysis according to PAM50 molecular subtype

**Fig. 3:**
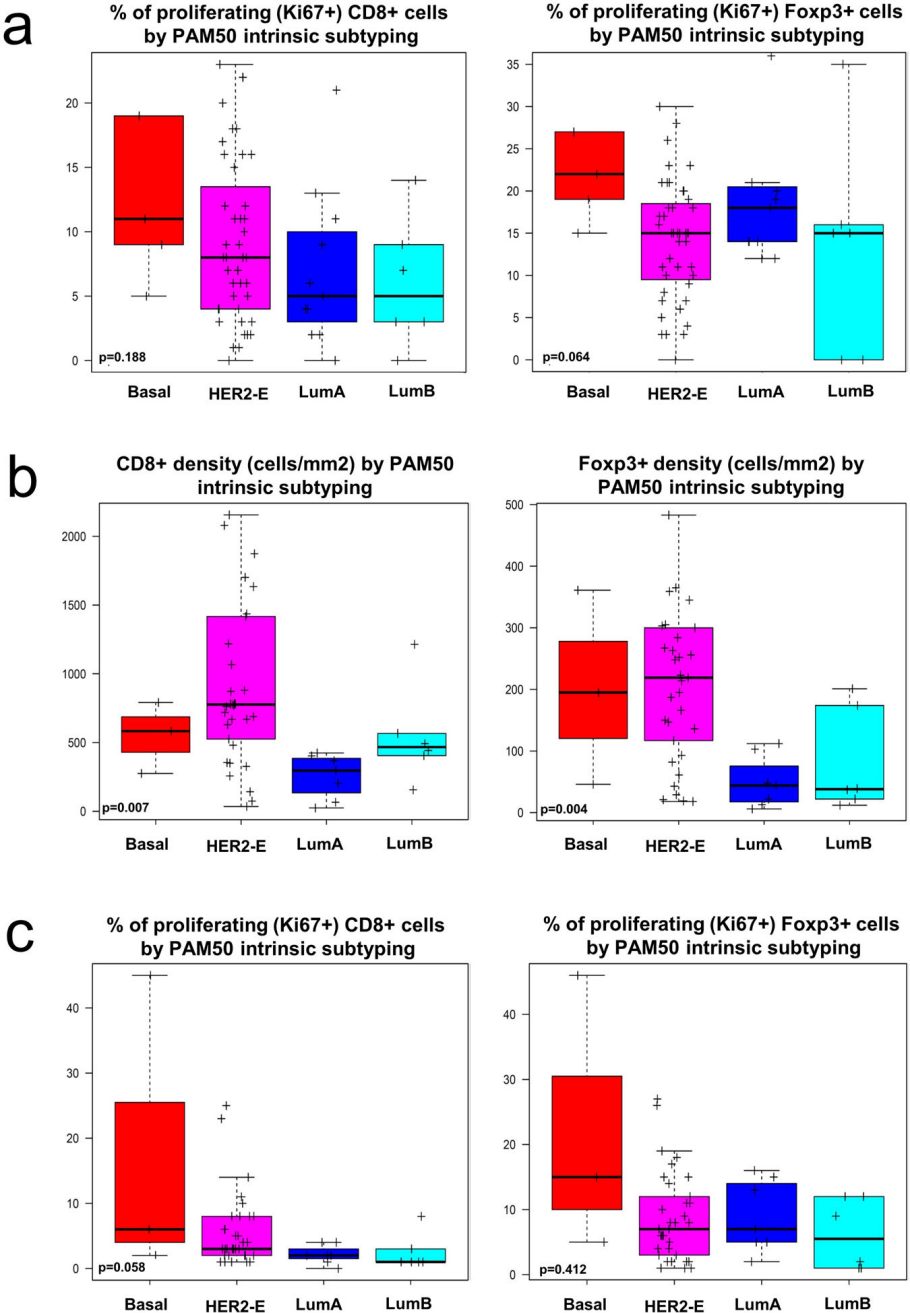
Immune cell density according to intrinsic subtype. **a** Boxplots of the proportion of proliferating immune cells (CD8^+^ and Foxp3^+^) at baseline according to baseline intrinsic subtyping. **b** Boxplots of immune cells densities (CD8^+^ and Foxp3^+^) at day 15 according to baseline intrinsic subtyping. **c** Boxplots of the proportion of proliferating immune cells (CD8^+^ and Foxp3^+^) at day 15 according to baseline intrinsic subtyping. Boxplot legend: centre line: median; bounds of box: interquartile range (IQR); whiskers: highest and lowest value excluding outliers (Q3 + 1.5*IQR to Q1 – 1.5*IQR); markers beyond the whiskers: potential outliers.

We have previously shown that, within HER2+ early BC, HER2-enriched PAM50 tumours present significantly higher sTILs as compared with other PAM50 subtypes, both at baseline (10% vs 5%; *P* = 0.006) and at day 15 (20% vs 10%; *P* < 0.001)^17^. Tumour-immune contexture analysis showed no significant difference in immune cell subsets densities according to PAM50 subtype at baseline (Table 1). The fraction of proliferating (Ki67^+^) cells for all four immune cell subpopulations (CD3^+^, CD4^+^, CD8^+^, Foxp3^+^) was numerically higher in basal-like tumours, whereas luminal tumours showed the lowest fraction of proliferating cells (Table 1, Fig. 3a, Supplementary Fig. 16a). However, this difference was not statistically significant.

**Table 1:** Immune cell density at baseline and after 2 weeks of anti-HER2 treatment according to baseline PAM50 intrinsic subtype.

Immune cell density at baseline					
Immune cell Subpopulation	Immune cell density by intrinsic subtype: median (IQR)				
	Luminal A (*N* = 11)	Luminal B (*N* = 6)	HER2-enriched (*N* = 43)	Basal-like (*N* = 5)	*p* value
CD3^+^	781 (200–1035)	1277 (696–1372)	1073 (367–2313)	644 (225–1921)	0.419
CD8^+^	217 (77–383)	461 (350–481)	475 (125–1018)	166 (64–648)	0.562
CD4^+^	352 (84–445)	525 (340–773)	507 (151–1096)	403 (144–502)	0.374
Foxp3^+^	43 (13–93)	28 (28–83)	88 (45–202)	52 (49–176)	0.178
%Ki67^+^CD3^+^	6 (4–7)	5 (3–8)	9 (4–14)	8 (5–17)	0.178
%Ki67^+^CD4^+^	6 (4–10)	7 (1–8)	9 (4–13)	9 (6–17)	0.184
%Ki67^+^CD8^+^	5 (2–9)	3 (3–7)	8 (4–12)	9 (5–19)	0.188
%Ki67^+^Foxp3^+^	18 (14–20)	15 (0–15)	15 (9–18)	19 (15–27)	0.064

Immune cell density at day 15					
Immune cell subpopulation	Immune cell density by intrinsic subtype: median (IQR)				
	Luminal A (*N* = 7)	Luminal B (*N* = 6)	HER2-enriched (*N* = 33)	Basal-like (*N* = 3)	*p* value
CD3^+^	435 (193–747)	749 (710–896)	1669 (899–2461)	1544 (501–1544)	**0.008**
CD8^+^	296 (66–367)	442 (405–492)	769 (481–1417)	583 (275–583)	**0.007**
CD4^+^	204 (102–322)	272 (228–295)	713 (361–1136)	629 (158–629)	**0.011**
Foxp3^+^	44 (13–48)	37 (22–39)	214 (93–300)	195 (46–195)	**0.004**
%Ki67^+^CD3^+^	3 (2–3	1 (1–1)	4 (2–7)	7 (3–7)	0.051
%Ki67^+^CD4^+^	3 (3–3)	1 (1–2)	5 (2–7)	10 (5–10)	0.062
%Ki67^+^CD8^+^	2 (1–2)	1 (1–1)	3 (2–8)	6 (2–6)	0.058
%Ki67^+^Foxp3^+^	7 (5–13)	2 (1–9)	7 (3–12)	15 (5–15)	0.412

Normal-like *N* = 0; significant *p* values in bold.

After 2 weeks of HER2-targeted treatment, tumours classified as HER2-enriched or Basal-like by PAM50 at baseline showed significantly higher density of all 4 immune subsets as compared to Luminal A and B tumours (Table 1, Fig. 3b, Supplementary Fig. 16b). Similar to baseline, the fraction of proliferating (Ki67^+^) cells for all four immune cell subpopulations (CD3^+^, CD4^+^, CD8^+^, Foxp3^+^) at day 15 was numerically higher in basal-like tumours, while luminal tumours showed the lowest fraction of proliferating cells, although the difference was not statistically significant (Table 1, Fig. 3c, Supplementary Fig. 16c).

### Immune contexture dynamics under anti-HER2 treatment

**Fig. 4:**
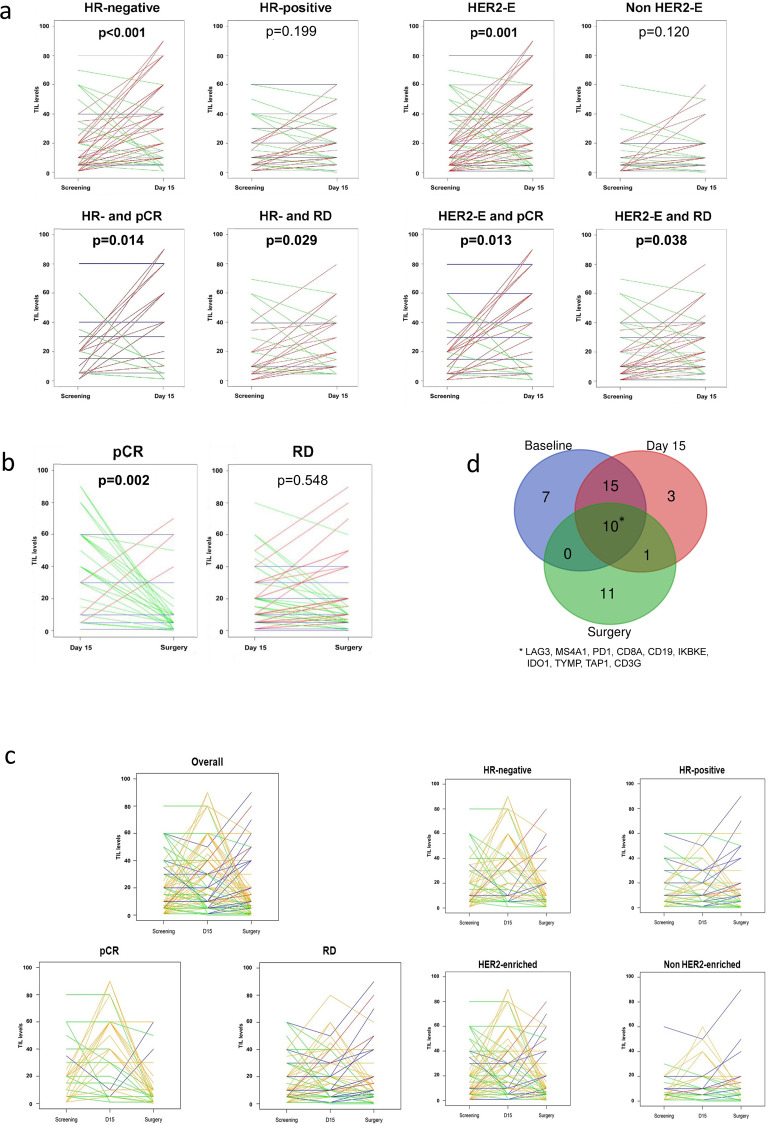
Changes in sTILs across timepoints and correlation with gene expression. **a** Changes in TILs between baseline and day 15 according to hormone receptor [HR] status, subtype (HER2-enriched [HER2-E]) and response (pathological complete response [pCR] vs residual disease [RD]). Lines are coloured according to TIL dynamics: increase (red), stable (blue) or decrease (green). **b** Changes in TIL levels between day 15 and surgery according to response: pathological complete response [pCR] vs residual disease [RD]. Lines are coloured according to TIL dynamics: increase (red), stable (blue), or decrease (green). **c** Changes in TIL levels between baseline, day 15 and surgery in the overall study cohort and according to response (pathological complete response [pCR] vs residual disease [RD]), hormone receptor [HR] status and PAM50 subtype. Lines are coloured according to TIL dynamics: increase between baseline and day 15 followed by an increase between day 15 and surgery (red); increase between baseline and day 15 followed by stable or decrease between day 15 and surgery (orange); stable or decrease between baseline and day 15 followed by an increase between day 15 and surgery (blue); stable or decrease between baseline and day 15 followed by stable or decrease between day 15 and surgery (green). **d** Venn diagram representing overlaps in genes upregulated in relation to increase in TIL levels across the three timepoints.

As previously described^17^, overall sTILs levels at day 15 were significantly higher than those in paired baseline samples. When we looked at differences according to subtype, a statistically significant increase in sTILs was observed in HR-negative (*P* < 0.001) and HER2-enriched subtype (*P* = 0.001), but not in HR-positive and non-HER2-enriched PAM50 subtypes (Fig. 4a, Table 2). Within the HER2-enriched subtype, an increase in sTILs levels was more evident in HR-negative disease (Table 2).

**Table 2:** Changes in TILs between day 15 and baseline according to PAM50 intrinsic subtype and hormone receptor (HR) status.

	*N* (pairs)	mean difference	95% confidence interval	*p* value
HR-positive	70	+2.1%	−0.7–+4.8	0.199
HR-negative	61	+12.5%	+5.5–+19.6	**<0.001**
HER2-enriched	85	+8.8%	3.5–14.0	**0.001**
HER2-enriched and HR−	53	+12.1%	4.3–20.0	**0.004**
HER2-enriched and HR+	32	+3.2%	−2.0–+8.4	0.235
Non-HER2-enriched	46	+3.5%	−0.3–+7.4	0.120
Non-HER2-enriched and HR−	8	+15.0%	−3.7–+33.7	0.106
Non-HER2-enriched and HR+	38	+1.1%	−1.6–+3.9	0.584
Basal-like	7	+11.6%	−8.6–+31.7	0.201
Normal-like	2	+9.5%	−365.3–+384.3	1.000
Luminal B	15	+3.9%	−1.7–+ 9.6	0.236
Luminal A	22	+0.18%	−2.16–+2.5	0.959

Significant *p* values in bold.

To identify which immune component was responsible for the increase in sTILs levels after priming with 2 weeks of anti-HER2 treatment, we analysed changes in the density of each immune cell subpopulation between these two timepoints by multiplexed spatial cellular phenotyping. We found large differences in both activity and densities of the immune cells when comparing untreated tumours with on-treatment samples. In fact, densities of all immune cells subtypes increased at day 15 [median (interquartile range) CD3^+^ 1462(1453), CD8^+^ 688(526), CD4^+^ 534(684), Foxp3^+^ 166(219)] as compared to baseline [median (interquartile range) CD3^+^ 832(1795), CD8^+^ 364(841), CD4^+^ 445(684), Foxp3^+^ 83(141)] tumours (*P* values, significant only for CD8^+^ = 0.04, Supplementary Table 1, Supplementary Fig. 13c). When individual patient immune cell density data from the 39 patients with paired baseline-day 15 samples were considered, a significant increase in both CD8^+^ and Foxp3^+^ cell density was observed at day 15 (Supplementary Fig. 6).

In on-treatment samples (day 15), 50% of all immune cells were CD8^+^ as compared with 42% of baselines samples (Fig. 2b). CD3^+^ and CD8^+^ immune cell densities were inversely correlated with tumour area in on-treatment (day 15) samples (Spearman’s rho, CD3^+^ −0.367 *P* = 0.020, CD8^+^ −0.368; *P* = 0.010) but not in baseline samples (CD3^+^ −0.060; *P* = 0.620, CD8^+^ −0.014; *P* = 0.915; Supplementary Fig. 8b–c).

Spatial analysis revealed that the increase in the number of immune cells at day 15 was significant in the intratumoural and proximal peritumoural regions but not in the distal stromal region (Supplementary Fig. 17). Upon treatment, the number of proliferating immune cells per area uniformly decreased across all location compared to baseline pretreatment samples (*P* < 0.001 for all comparisons, Supplementary Figs. 13d and 18, Supplementary Table 5). Lower percentages of proliferating immune cells at day 15 were not significantly associated with lower tumour cellularity evaluated on the same sample (Spearman Rho, CD3^+^Ki67^+^ = 0.089, CD8^+^Ki67^+^ = 0.053, CD4^+^Ki67^+^ = 0.185, Foxp3^+^Ki67^+^ = 0.246, *P* > 0.05 for all comparisons) (Supplementary Fig. 11b, c).

Paired multiplex IHC (mIHC) data from both baseline and day 15 samples were available from 39 patients and were used to assess changes in densities of immune cell subpopulations in day 15 and baseline paired samples according to baseline PAM50 intrinsic subtype and HR status (Supplementary Table 6). Although the decrease in percentages of proliferating immune cells (all four immune subpopulations) at day 15 was consistently observed across all subgroups, a statistically significant increase in immune cells density (all four immune subpopulations) at day 15 was only observed in HR-negative and HER2-enriched subtype (all *P* values < 0.05 except for CD4+in HER2-enriched tumours *P* = 0.055), but not in HR-positive and non-HER2-enriched PAM50 subtypes. Similar to prior observations of sTILs levels, the increase was numerically more evident in HR-negative disease than in HER2-enriched tumours.

### Tumour-immune contexture analysis and the probability of achieving a pCR

As previously described^17^, higher sTILs were significantly associated with pCR and lower residual cancer burden scores, both at baseline and after 2 weeks of anti-HER2 treatment.

In HR-positive disease or non-HER2-enriched subtype, no consistent change in sTILs at day 15 versus baseline was found according to the type of pathological response (Supplementary Table 7). Both in HER2-enriched subtype and HR-negative disease, a consistent increase in sTILs at day 15 versus baseline was found regardless of the type of pathological response (Fig. 4a and Supplementary Table 7).

**Fig. 5:**
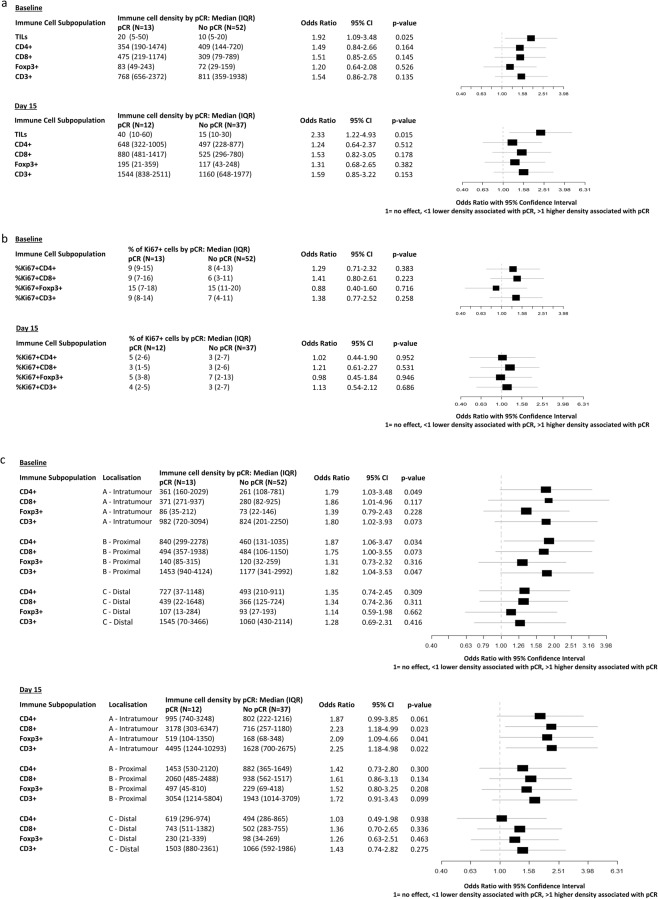
Immune cell density and pathological complete response. **a** Odds ratios (95% confidence interval) for pathologic complete response (pCR) for 10% increases in TIL levels and 1000 cells/mm^2^ increases in immune cell density evaluated on baseline and Day 15 (on-treatment) samples. **b** Odds ratios (95% confidence interval) for pathologic complete response (pCR) for increases in % of proliferating immune cells for each immune cell subpopulation evaluated on baseline and day 15 (on-treatment) samples. **c** Odds ratios (95% confidence interval) for pathologic complete response (pCR) for 1000 cells/mm^2^ increases in immune cell density according to immune cell localisation evaluated on baseline and day 15 (on-treatment) samples.

To identify if the prognostic impact of sTILs might be different according to immune contexture, we then analysed the impact of immune cells composition, activity and spatial interaction with tumour cells on pCR. Both at baseline and at day 15, no significant difference in immune cell subpopulation densities was observed between tumours achieving or not achieving pCR, despite numerically higher densities of all four immune subpopulations were observed in tumours achieving pCR at day 15 (Fig. 5a).

After that, to determine whether T-cell activation status associated with the probability of pCR, we compared the rates of proliferating (Ki67^+^) immune cells in tumours achieving or not achieving pCR. No statistically significant association was observed, although Odds ratios (OR) for % of proliferating cells and pCR were consistently higher at baseline as compared with day 15 for all immune cell subpopulations except Foxp3^+^ (Fig. 5b).

Finally, we analysed immune cells subpopulation densities separately according to their spatial distribution. Although only some of the associations reached statistical significance, the association between higher immune cell density and pCR was consistently stronger for more proximal compartments (intratumoural and proximal peritumoural stroma) as compared with the distal stroma compartment for all four immune cell subpopulations. Moreover, the association between higher immune cell density and pCR was stronger at day 15 as compared with baseline (Fig. 5c).

### Changes in sTILs between day 15 and surgery

At surgery, median sTILs levels were 10% (quartile 1–3: 5–20). Paired sTILs data from day 15 and surgery samples were available for 124 patients (82.1%). An increase and a decrease of sTILs between day 15 and surgery were observed in 26.4% (24/91) and 33.0% (30/91) of tumours, respectively, whereas for 40.7% (37/91) of tumours the same percentage of sTILs was reported at day 15 and at surgery. Compared with day 15, a significant decrease in sTILs was observed at surgery in tumours achieving a pCR (mean difference −21.5%, 95% CI −33.3 to −9.7, *P* = 0.002, Fig. 4b), but not in patients with residual disease at time of surgery (mean difference −0.9%, 95% CI −4.1–+2.4, *P* = 0.548, Fig. 4b). 89.7% of residual tumours (non-pCR) had sTILs above ≥5%. The distribution of residual tumours (non-pCR) according to sTILs at surgery was 10.3% (sTILs < 5%), 38.1% (sTILs 5–9%), 22.7% (sTILs 10–19%), 16.5% (sTILs 20–39%) and 12.4% (sTILs ≥ 40%). Distribution of tumour samples (non-pCR and pCR) according to sTIL levels at the three timepoints is presented in Supplementary Tables 8–9 and Supplementary Fig. 19. A decrease in sTILs in tumours achieving pCR was observed irrespectively of HR status and intrinsic subtype (Supplementary Table 10). In tumours not achieving a pCR, no significant tendency was observed. Finally, TILs at surgery were not found statistically significantly different according to the type of pathological response (median sTIL levels at surgery 10% (5–20) vs 5% (1–10) in patients with residual disease and achieving pCR, respectively; *P* = 0.662).

### sTILs dynamics across timepoints

Different tumour-infiltrating lymphocytes dynamics observed across the three timepoints (*N* = 122 patients with sTILs data from all three timepoints) are recapitulated in Supplementary Table 11 and Fig. 4c. The most frequently observed pattern (*N* = 32, 26%) was an increase in TILs from baseline to day 15 followed by a decrease from day 15 to surgery. This pattern was observed especially in HR-negative tumours, HER2-enriched tumours and tumours achieving pCR (Fig. 4c). Changes in sTILs levels between surgery and baseline paired samples according to the achievement of pCR, HR status and PAM50 subtypes are reported in Supplementary Table 12.

### sTILs vs gene expression

To evaluate genes associated with sTILs, we explored data from 413 samples with paired gene expression and sTIL data (from all three timepoints mixed: baseline *N* = 148; day 15 *N* = 133; surgery *N* = 132). A total of 555 BC-related genes were evaluated, including 72 immune-related genes (Supplementary Table 13). Using a quantitative significance of microarrays (SAM) analysis, 36 upregulated genes were found associated with sTIL levels (false discovery rate (FDR) < 1%) (Supplementary Table 14), the top upregulated gene being MS4A1 (CD20). Functional annotation of the 36 genes using DAVID annotation tool^19^ revealed that 50% of them were significantly involved in immune response (e.g., CD3G, CD8A, CD4 and LAG3) and regulation of the immune system process (e.g., IDO1, IL6R, STAT1 and PD1), 33% of them were involved in lymphocyte activation (e.g., CD84, CD86, CD3G and CD4) and 28% of them involved in T-cell activation (e.g., CD8A, PD-L1, RELB and CD4).

When a similar analysis was performed within each timepoint separately, similar results were obtained (Fig. 4d; Supplementary Tables 15–17). Among the different genes significantly associated with sTIL levels, 10 (MS4A1, PD1, CD8A, CD19, IKBKE, IDO1, TAP1, TYMP, CD3G and LAG3) were found consistently associated with sTILs across all timepoints. This 10-gene list was found highly enriched (FDR < 1%) for immune genes tracking activated CD8 T cells (e.g., CD8A, CD3G, LAG3, PD1). The correlation coefficients of the expression of these genes with baseline sTILs ranged from 0.52 in gene CD8A to 0.34 in gene TYMP.

## Discussion

To our knowledge, our report is the first one to provide new insights into TIL variations and immune contexture during HER2-targeted therapy in the absence of chemotherapy. Moreover, using a novel mIHC technique, immune infiltrate at baseline and after 2 weeks of anti-HER2 treatment was characterised in its immune cell subpopulations and analysed according to proximity to tumour cells and activity (using co-expression of Ki67 marker to identify proliferating immune cells).

First, in early HER2+ treatment-naive BC, tumour-immune contexture analysis showed no significant difference in immune cell subsets densities according to intrinsic subtyping. However, we observed a significantly higher proportion of proliferating immune cells in HR− as compared with HR+ tumours, except for Foxp3^+^ and consistently observed a numerically higher percentage of proliferating immune cells in basal-like tumours and HER2-enriched tumours and a numerically lower percentage of proliferating immune cells in luminal tumours. However, it should be pointed out that the very limited number of non-HER2-enriched tumours identified in the PAMELA trial, in line with what expected in early HER2+ BC, significantly limits the power of these analyses.

Second, after 2 weeks of dual HER2-targeted therapy, a general increase in sTILs is observed. However, this increase appears to be selectively present in HR-negative and HER2-enriched subtype, regardless of pathological response at surgery, but not in HR-positive and non-HER2-enriched subtypes. Immune contexture analysis highlighted that this increase is not linked to a selective increase of one immune cell subpopulation, but a concomitant increase in cell density of all four immune subpopulations (CD3+, CD4+, CD8+, Foxp3+) after anti-HER2 treatment. However, a significant shift was observed for CD8+ cytotoxic T cells subpopulation, which represented 50% of all tumour-associated immune cells in on-treatment samples.

As previously observed for sTIL levels, increase in specific immune cell subpopulation densities was observed in HER2-enriched subtype and HR-negative tumours, but not in non-HER2-enriched PAM50 subtypes and HR-positive tumours.

Consistently with these trends, immune infiltrate was radically modified after 2 weeks of anti-HER2 treatment. In fact, after priming with anti-HER2 treatment, tumours that were HER2-enriched at baseline showed higher densities of all four immune cell subpopulations, highlighting the differential activation of the immune system towards the disease after priming with anti-HER2 treatment according to tumour biology.

Moreover, the association between pCR and immune infiltrate was stronger at day 15 than at baseline, both for sTILs and specific immune subpopulations, especially when immune cells intratumour/more proximal to the tumour were considered, pointing out the potential biological role of immune activation after anti-HER2 priming in early HER2+ BC.

Subsequently, a general decrease in sTIL levels is observed at surgery. However, this decrease is driven by tumours achieving pCR, whereas no significant trend was seen in patients with residual disease at the time of surgery. Tumours achieving pCR are characterised by an increase in sTIL levels after 2 weeks of anti-HER2 treatment and a decrease in sTIL levels at surgery. This might be linked to downregulation of immune response after clearing tumour cells. However, although the increased infiltration of immune cells observed after HER2 priming is inversely correlated with tumour cellularity at day 15, hinting that these immune cells might have been actively clearing tumour cells during the first 2 weeks of HER2-targeted treatment, their activity (in terms of fraction of proliferating immune cells) was significantly and homogeneously reduced after 2 weeks of HER2-targeted treatment, independently for the amount of residual tumour in the sample and tumour characteristics, thus pointing out that immune exhaustion processes might already be at work at this early timepoint. This observation might by relevant to address the question of which might be the ideal timing of potential combination with immunotherapy in early HER2+ BC.

The association between pCR and decrease in sTIL levels at surgery has also been shown after chemotherapy-containing neoadjuvant treatment for HER2+ BC^12^. However, the same study observed that higher sTIL levels at surgery, in presence of residual disease, were associated with an adverse disease-free survival, suggesting that post-neoadjuvant sTILs might be unable to exert their antitumour function, possibly owing to an immunosuppressive microenvironment or T-cell exhaustion. A limitation of the present study is that immune infiltrate subtyping by mIHC was not available for surgical samples with residual disease and therefore functional assessment of this infiltrate could not be evaluated. Moreover, the true prognostic value of sTIL levels after dual HER2 blockade without chemotherapy remains unknown and long-term follow-up data from the PAMELA trial is not currently available to provide more information on this point.

However, the presence of high sTIL levels in most residual tumours at surgery might imply that these patients might be good candidates for clinical trials evaluating adjuvant immune checkpoint inhibitors. The KATE2 trial, which tested the addition of the anti-PD-L1 antibody atezolizumab to trastuzumab emtansine in metastatic BC HER2+ BC patients previously treated with trastuzumab and taxanes, despite missing its primary endpoint, identified a numerically longer PFS and higher 1-year OS in patients with PD-L1+ and TIL high (≥5%) tumours^20^. These hypothesis-generating data might support the evaluation of PD1/PD-L1 inhibitors to trastuzumab emtansine in the post-neoadjuvant setting to further improve the prognosis of patients with inflamed residual disease.

Tumour heterogeneity has a predominant role in modulating immune activation in HER2+ BC. Indeed, not only HR-negative and HER2-enriched tumours had higher sTIL levels at baseline, but the impact of tumour biology was observed even more clearly after exposure to HER2-targeted treatment. Non-luminal subtypes showed the highest increases in sTIL levels between baseline and day 15, whereas luminal subtypes showed modest/no increase. Even within HER2-enriched tumours, an increase in sTILs was predominantly seen in HR-negative tumours rather than in the HR-positive subgroup. Indeed, HR positivity appeared to be associated with the capacity/incapacity of HER2+ BC to inflame during dual HER2 blockade (without chemotherapy), more than to baseline sTIL levels. These observations were also supported by immune contexture analyses showing a significantly lower proportion of CD8^+^Ki67^+^ T cells and higher ratio of proliferating Foxp3^+^/CD8^+^ in HR+ as compared with HR- tumours. Whether this is due to specific regulation of the immune system by hormone signalling (or endocrine treatment, as all HR-positive BCs also received hormonotherapy in the PAMELA trial), or if reduced activation of immunity in these tumours is linked to reduced cell death after HER2-targeted treatment and reduced antigen exposure, remains unclear and might hopefully be the subject for further investigation.

In conclusion, in early HER2+ BC, an increase in sTIL levels is observed following 2 weeks of dual HER2 blockade, in HR-negative disease and HER2-enriched subtype. Immune contexture analysis revealed that the strongest impact on pCR was achieved when immune cells spatially interacted with tumour cells. Afterward, sTILs consistently decreased at surgery in patients achieving a pCR, whereas most residual tumours at surgery remained inflamed, possibly reflecting a progressive loss of function of T cells, which is already evident after 2 weeks of treatment. Understanding the features of the resulting tumour immunosuppressive microenvironment has crucial implications for the success of checkpoint blockade and adoptive T-cell transfer therapies. Beyond modulating baseline immune activation, tumour biology also has a role in modulating the dynamic activation of the immune system after exposure to HER2-targeted treatment. This should be taken into account as the role of immunity and immunotherapy is further assessed in HER2+ BC.

## Methods

### PAMELA clinical trial

The main results of the neoadjuvant PAMELA phase II trial (NCT01973660) have been previously reported^15^. In this study, 151 early HER2+ BC patients were treated with the combination of lapatinib (1000 mg daily) and trastuzumab (8 mg/kg i.v. loading dose followed by 6 mg/kg) for 18 weeks. Patients with HR-positive disease also received letrozole or tamoxifen according to menopausal status (Supplementary Fig. 1a). In the PAMELA trial, tissue collection was mandatory as it was used for primary endpoint determination. Tumour samples were collected at three timepoints according to the protocol: baseline (within 28 days preceding treatment start), day 15 (a ± 5 days window was admitted, but collection of samples the closest as possible to preplanned timepoint was warmly suggested) and surgery (Supplementary Fig. 1a and sample flow by REMARK diagram in Supplementary Fig. 1b). A minimum of 2 core formalin-fixed paraffin-embedded (FFPE) samples were collected by tru-cut biopsy at each timepoint (except at surgery). In case of multifocality, samples were collected from the same lesion. Samples were primarily used for pre-specified protocol analyses, which included central HER2, ER and PR confirmation by regular IHC, Ki67 by IHC and molecular subtyping by PAM50 gene expression assays. The mIHC analyses performed in the present study were post-hoc and used left-over samples.

The PAMELA trial was conducted under Good Clinical Practice guidelines and the Declaration of Helsinki. The study protocol was approved by independent ethics committees at each centre (trial centres listed at clinicaltrials.gov, NCT01973660). All patients provided written informed consent.

### sTILs evaluation

Stromal TILs at baseline, day 15 and surgery were centrally evaluated on whole sections of tumour tissue stained with H&E blinded from clinical-pathological and outcome data. Percentages (%) of TILs at baseline and day 15 were scored in slides of core biopsies. sTILs were quantified according to the 2014 Guidelines developed by the International TILs Working Group^18,21^. The reproducibility of this method has been described previously^2^.

### Multiplex IHC (NGI)

Baseline (*N* = 65) and day 15 (*N* = 49) biopsies from 75 patients were analysed using a custom mIHC 6-plex panel, based on iterative cycles of staining and destaining of the same slide with different primary antibodies, individual slide digitalisation, virtual multiplexed digital image reconstruction and complex image analyses.

Before cutting, FFPE blocks were cooled to −10 °C and 3 μm sections were cut with a microtome. Sections were collected on positively charged Superfrost glass slides and dried overnight at 37 °C. The first IHC staining was performed (information of all the protocols on Supplementary Table 18) in Discovery Ultra Autostainer (Ventana Medical Systems, Tucson AZ). Mono AEC/Plus (#K050; PALEX) was used as the chromogen.

The slides were mounted with aqueous-based mounting medium. The stained slides were digitalised at 20× using the NanoZoomer 2.0HT (Hamamatsu Photonics, Japan). After digitalisation, coverslips were taken and slides were put in increasing alcohol solutions until 100% and then in decreasing alcohols until water before slides were loaded in the Discovery Ultra autostainer for the next immunostaining.

Any residual primary antibody was stripped by an ‘extra' antigen retrieval step before the staining process is repeated for the following primary antibody. Heat-induced antigen retrieval was done using ULTRA Cell Conditioning 2 (ULTRA CC2, Ventana Medical Systems, Tucson AZ) for 8 minutes at 100 °C and DISCOVERY Cell Conditioning 1 (DISCOVERY CC1, Ventana Medical Systems, Tucson AZ) for 40 minutes at 95 °C to block the previous antibody and the process was repeated consecutively six times.

To avoid primary antibody cross-reactivity between cycles owing to incomplete stripping, we used several strategies. First, we added an extra antigen retrieval step before the next cycle of staining to prevent any remnant reactivity to primary or secondary antibodies used in the first cycle. Second, the protocol alternated rabbit and mouse primary antibodies to reduce cross-reactivity. Third, the sequence of primary antibodies alternated nuclear (Ki67 and Foxp3), membrane (CD3, CD8, CD4) and cytoplasmic (CK) markers (Supplementary Fig. 20).

The sequential staining procedure was automatised, thus significantly reducing the hands-on time (1-hour per staining cycle) and duration of the entire process (3 days per 6-plex panel run in 30 slides).

The image analysis pipeline was the following one: first, images were uploaded into VISIOPHARM® (VIS) Image Analysis Software (Visiopharm Integrator System version 2019.02.1.6005, Visiopharm, Denmark) for registration. Images were automatically aligned and fused into a single virtual digital image (VDI) using the Tissuealign® module of VIS (order of alignment: Foxp3, CD3, CD8, CD4, Ki67, cytokeratin). After alignment, images were analysed with custom-developed algorithms created using the Author® module of VIS (algorithms in Supplementary Material).

After this, we performed automatic tissue recognition of the aligned slides and the selected areas were reviewed by a pathologist, who manually defined the ROI, which included the tumour and surrounding peritumoural stroma (the tumour bed in case of complete regression) and excluded normal and/or necrotic area. Following this, we ran T-cell density APP was run on the entire slides to obtain global results. The T-cell application (detailed in Supplementary Table 19) uses a cell classification method based on form and size and a pixel-colour intensity threshold method to classify the cells into Foxp3, CD3, CD4, CD8 cells on one hand, and uses the Ki67 staining to inform about the percentage of the cell populations that are proliferating on the other hand. Any brown stained nucleus was considered a positive cell.

We then applied a third APP (location APP), which uses the PANCK staining to divide the ROI created by the pathologist into three different ROIs: the tumour area (A), the stroma within 30 µm from tumour (B) and the stroma >30 µm from tumour (C). For that purpose, we used HDAB-DAB feature, which enhances the brown staining corresponding to the PANCK staining (A). Dilation was used to create B and C ROIs. After applying the location APP to the data set, we used the T-cell APP to obtain densities and proliferation rates of different cell populations across different locations.

Created APPs were trained by a biotechnologist expert in image analysis and the results validated by a board-certified pathologist. Data were finally reported as densities of each category of cells in the tumoural area in general, for each location, and the proliferation rate of each of the cell categories.

### Gene expression analysis

Samples from all three timepoints were analysed using the same methodology. First, a section of FFPE breast tissue was examined with H&E staining to confirm the diagnosis and determine the tumour surface area. RNA purification was performed after macrodissection, when needed, to avoid normal breast contamination. RNA was extracted from FFPE material using the High Pure FFPET RNA isolation kit (Roche, Indianapolis, IN, USA) following the manufacturer’s protocol. RNA samples were quantified at the NanoDrop spectrophotometer (Thermo Fisher Scientific, Waltham, MA, USA).

A minimum of ~100 ng of total RNA was used to measure the expression of 555 BC-related genes and five housekeeping genes (ACTB, MRPL19, PSMC4, RPLP0 and SF3A1) using the nCounter platform (Nanostring Technologies; Seattle, Washington, USA^22^). Data were log base 2–transformed and normalised using the housekeeping genes. The complete list of genes, which included immune-related genes (e.g., CD8A, CD4, PD1 and PD-L1), can be found in Supplementary Table 13. Intrinsic molecular subtyping at baseline was determined using the previously reported PAM50 subtype predictor^23^.

### Statistical analysis

Spearman test was used for correlation analysis and Mann–Whitney *U* test and Wilcoxon test were used for all the density, location and proliferation analyses. To determine differences in the distribution of TIL levels or immune cell density across subgroups Mann–Whitney *U* and Kruskal–Wallis test were used according to number of subgroups. Significant changes in sTILs or immune cell density between two timepoints were determined using paired Wilcoxon tests. The association of each variable with pCR was determined by univariate logistic regression analysis. As per study protocol, pCR was defined as the absence of residual invasive cancer in the breast following neoadjuvant therapy (ypT0/is). OR with a 95% confidence interval were estimated. All statistical tests were two-sided and considered significant when *p* < 0.05.

When recapitulating TIL dynamics across the three timepoints, any increase/decrease in sTIL levels were taken into account and sTILs were only defined as unchanged if the same % of TILs were present at two subsequent timepoints.

To identify genes whose expression was significantly different according to sTIL levels as a continuous variable, we used a quantitative SAM analysis with an FDR < 1%. Pearson correlations were used to evaluate the association of expression of a single gene with sTILs expression. Biologic analysis of gene lists was performed with DAVID annotation tool (http://david.abcc.ncifcrf.gov/)^19^.

All statistical analyses were performed using the R software 3.6.1.

### Reporting summary

Further information on research design is available in the Nature Research Reporting Summary linked to this article.

## Supplementary information

Supplementary Material

REPORTING SUMMARY

## Data Availability

The data generated and analysed during this study are described in the following data record: 10.6084/m9.figshare.13681456^24^. The data files underlying the related study are available from the corresponding authors upon reasonable request. However, several files are not publicly available in order to protect patient privacy. A comprehensive list of data files underlying the related manuscript along with details of their availability is contained in the spreadsheet ‘Griguolo_et_al_2021_underlying_datafile_list.xlsx’, available as part of the figshare. The custom-developed algorithms (T-cell APP) created using the Author® module of VISIOPHARM® (VIS) Image Analysis Software (Visiopharm Integrator System version 2019.02.1.6005, Visiopharm, Denmark) are also available as part of the figshare data record.

## Code availability

R codes used for data analysis during this study are available from the corresponding authors on reasonable request.
