# Supplementary material for: Immune microenvironment characterisation and dynamics during anti-HER2-based neoadjuvant treatment in HER2-positive breast cancer
Source: NPJ Precis Oncol. 2021 Mar 19;5:23. doi: 10.1038/s41698-021-00163-6 (PMC7979716; doi:10.1038/s41698-021-00163-6)
Supplement: Supplementary file 1 — Supplementary Material [file 41698_2021_163_MOESM1_ESM.pdf]

Supplementary Table 1. Immune cells densities

	Timepoint	n	mean	median	min	max	IQR	p value (SCR vs D15)
**CD3**	ALL	114	1498	1169	33	7278	1650	
	SCR	65	1379	832	33	5579	1795	
	W2	49	1655	1462	140	7278	1453	0.14
**CD8**	ALL	114	733	487	18	4653	763	
	SCR	65	638	364	18	2975	841	
	W2	49	859	668	24	4653	526	0.04
**CD4**	ALL	114	629	500	12	2488	719	
	SCR	65	608	445	12	2402	684	
	W2	49	658	534	48	2488	684	0.25
**Foxp3**	ALL	114	161	108	0	1460	190	
	SCR	65	133	83	0	844	141	
	W2	49	198	166	6	1460	219	0.05

Supplementary Table 2. Proportion of proliferating T cells by location (ALL dataset)

CD3+Ki67+							
**LOCATION**	**n**	**mean**	**median**	**min**	**max**	**IQR**	**pvalue**
**A**	**111**	11.9	**9.72**	0	60.4	12.7	
**B**	**110**	7.74	**6.30**	0	33.6	6.99	
**C**	**110**	4.50	**3.48**	0	18.3	4.04	<0.0001
**CD4+Ki67+**							
**A**	**111**	12.7	**11.0**	0	60.3	13.2	
**B**	**110**	8.63	**7.43**	0	33.4	7.48	
**C**	**110**	6.18	**5.39**	0	21.0	5.57	<0.0001
**CD8+Ki67+**							
**A**	**111**	11.9	**8.74**	0	60.7	13.0	
**B**	**110**	7.85	**5.68**	0	35.3	7.59	
**C**	**110**	5.57	**4.29**	0	34.5	5.17	<0.0001
**Foxp3+Ki67+**							
**A**	**109**	18.3	**15.9**	0	85.7	18.2	
**B**	**110**	13.7	**13.3**	0	50	13.7	
**C**	**110**	10.5	**9.28**	0	100	9.85	<0.0001

Supplementary Table 3. Differences of immune cells by HR status across locations

	**CD3**						
	HR status	mean	median	min	max	IQR	Wilcoxon
A	HR-	3324.	1665.	19.4	20234.	2686.	
	HR+	1833.	1235.	48.6	17819.	1779.	0.2
	HR status	mean	median	min	max	IQR	
B	HR-	3285.	2949.	68.8	13498.	3199.	
	HR+	1829.	1261.	38.2	11628.	2143.	0.005
	HR status	mean	median	min	max	IQR	
C	HR-	1933.	1760.	0	6748.	2004.	
	HR+	1256.	884.	40.7	4945.	1099.	0.012
	**CD4**						
	HR status	mean	median	min	max	IQR	
A	HR-	1108.	722.	3.95	6095.	1309.	
	HR+	711.	413.	19.1	4182.	861.	0.3
	HR status	mean	median	min	max	IQR	
B	HR-	1205.	890.	31.2	4017.	1632.	
	HR+	766.	530.	8.97	3503.	794.	0.012
	HR status	mean	median	min	max	IQR	
C	HR-	791.	711.	0	2524.	876.	
	HR+	551.	424.	18.8	2037.	574.	0.038
	**CD8**						
	HR status	mean	median	min	max	IQR	
A	HR-	1938.	668.	11.9	15641.	1863.	
	HR+	929.	585.	15.2	12010.	777.	0.19
	HR status	mean	median	min	max	IQR	
B	HR-	1782.	1150.	32.6	10111.	1875.	
	HR+	847.	562.	22.7	6744.	865.	0.004
	HR status	mean	median	min	max	IQR	
C	HR-	911.	663.	0	4153.	1146.	
	HR+	540.	366.	16.3	2863.	455.	0.011
	**Foxp3**						
	HR status	mean	median	min	max	IQR	
A	HR-	348.	161.	0.359	2756.	373.	
	HR+	191.	90.1	0	1743.	137.	0.085
	HR status	mean	median	min	max	IQR	
B	HR-	380.	292.	4.13	2521.	422.	
	HR+	194.	97.7	0.406	1819.	206.	0.00036
	HR status	mean	median	min	max	IQR	
C	HR-	225.	216.	0	1412.	238.	
	HR+	122.	70.0	0.646	999.	123.	0.0013

Supplementary Table 4. Proportion of proliferating T cells by HR status

	SUBTYPE	n	mean	median	min	max	IQR	p value
CD4	HR-	61	9.80	8.74	0.530	41.4	8.38	
	HR+	53	6.21	5.35	0	21.7	5.74	0.0088
CD8	HR-	61	9.55	6.93	0.410	50.5	9.52	
	HR+	53	5.43	4.02	0	22.6	6.15	0.011
Foxp3	HR-	61	13.6	13.5	1.03	46.3	11.6	
	HR+	53	13.2	12.1	0	75	12.8	0.71
CD3	HR-	61	9.39	7.77	0.472	43.6	8.20	
	HR+	53	5.58	4.57	0	22.4	5.15	0.0065
Foxp3/CD8	HR-	61	1.90	1.33	0.644	6.43	1.46	
	HR+	51	3.67	2.29	0	19.6	2.37	0.0039

Supplementary Table 5. Immune cells proliferation by timepoint

	TIMEPOINT	n	mean	median	min	max	IQR
**CD4+Ki67+**	SCR	65	10.0	8.81	0	40.2	10.3
	W2	49	5.62	3.67	0.651	41.4	4.23
**CD8+Ki67+**	SCR	65	9.30	7.60	0	50.5	8.53
	W2	49	5.42	3.03	0.348	44.7	3.85
**Foxp3+Ki67+**	SCR	65	16.4	15.0	0	75	9.54
	W2	49	9.34	7.48	0.796	46.3	10.0
**CD3+Ki67+**	SCR	65	9.28	7.90	0	43.6	9.47
	W2	49	5.43	3.16	0.685	42.5	4.57

Supplementary Table 6. Changes in densities of immune cell subpopulations between Day 15 and baseline according to baseline PAM50 intrinsic subtype and hormone receptor (HR) status

Immune Cell Population	Day 15: Median (IQR)	Baseline: Median (IQR)	N (pairs)	mean difference	95% CI	p-value
Overall (N=39)						
CD3+	1312 (721-2203)	781 (493-2061)	39	496	-13 - + 1004	0.135
CD8+	669 (352-884)	350 (135-798)	39	372	67 - 678	**0.026**
CD4+	532 (282-1014)	354 (202-664)	39	129	-71 - 329	0.171
Foxp3+	166 (45-374)	80 (29-156)	39	101	37- + 166	**0.002**
%Ki67+CD3+	3 (2-7)	8 (4-11)	39	-3.7	-5.4 - -2.0	**<0.001**
%Ki67+CD8+	3 (2-6)	7 (4-11)	39	-3.7	-5.7 - -1.8	**<0.001**
%Ki67+CD4+	3 (2-7)	9 (4-14)	39	-4.3	-5.9 - -2.6	**<0.001**
%Ki67+Foxp3+	8 (3-14)	15 (10-19)	39	-6.0	-8.8 - - 3.2	**<0.001**
HR-positive (N=17)						
CD3+	749 (433-925)	781 (569-1372)	17	-102	-560 - 355	0.517
CD8+	367 (156-566)	350 (115-786)	17	31	-201 - + 263	0.795
CD4+	295 (213-527)	352 (213-720)	17	-108	-316 - 100	0.225
Foxp3+	48 (29-117)	43 (28-110)	17	26	-22 - + 74	0.218
%Ki67+CD3+	3 (1-4)	5 (3-8)	17	-3.3	-5.6 - -1.0	**0.003**
%Ki67+CD8+	3 (1-4)	4 (3-8)	17	-3.3	-6.2 - -0.4	**0.035**
%Ki67+CD4+	3 (2-5)	7 (3-9)	17	-3.3	-5.6 - -0.9	**0.009**
%Ki67+Foxp3+	7 (4-12)	15 (9-18)	17	-6.4	-11.3 - -1.4	**0.022**
HR-negative (N=22)						
CD3+	1892 (1358-2397)	799 (466-2130)	22	958	148 - 1768	**0.033**
CD8+	779 (668-1281)	333 (146-926)	22	636	133 - 1139	**0.010**
CD4+	839 (489-1160)	382 (198-559)	22	312	3 - 621	**0.023**
Foxp3+	250 (159-305)	108 (48-187)	22	159	54 - 265	**0.003**
%Ki67+CD3+	6 (3-8)	10 (8-16)	22	-4.0	-6.6 - -1.5	**0.004**
%Ki67+CD8+	5 (2-8)	9 (6-15)	22	-4.1	-6.9 - -1.3	**0.002**
%Ki67+CD4+	6 (2-10)	10 (8-16)	22	-5	-7.4 - -2.6	**<0.001**
%Ki67+Foxp3+	9 (2-14)	15 (10-20)	22	-5.7	-9.2 - -2.1	**0.004**
Non HER2-Enriched (N=12)						
CD3+	624 (431-903)	807 (580-1196)	12	-134	-716 - 448	0.339
CD8+	332 (191-445)	328 (106-683)	12	-44	-346 - + 258	0.791
CD4+	250 (150-348)	381 (331-508)	12	-47	-296 - 202	0.339
Foxp3+	42 (20-64)	40 (24-90)	12	12	-27 - 51	0.583
%Ki67+CD3+	3 (2-3)	5 (3-7)	12	-2.1	-3.7 - -0.4	**0.024**
%Ki67+CD8+	2 (1-4)	4 (3-7)	12	-2.2	-4.3 - +0.1	0.051
%Ki67+CD4+	3 (2-5)	6 (2-9)	12	-1.9	-4.3 - + 0.5	0.298
%Ki67+Foxp3+	11 (4-15)	15 (12-21)	12	-5.5	-12.0 - + 1.0	0.182
HER2-Enriched (N=27)						
CD3+	1780 (1210-2412)	768 (392-2226)	27	776	93- + 1458	**0.041**
CD8+	777 (578-1318)	360 (158-872)	27	557	144 - 670	**0.004**
CD4+	713 (452-1142)	354 (190-911)	27	207	-65 - 479	0.055
Foxp3+	223 (142-304)	88 (41-183)	27	141	52- + 230	**0.002**
%Ki67+CD3+	5 (2-8)	8 (5-15)	27	-4.4	-6.8 – 2.1	**<0.001**
%Ki67+CD8+	4 (2-8)	8 (6-14)	27	-4.4	-7.2 - -1.7	**0.001**
%Ki67+CD4+	5 (2-8)	9 (7-16)	27	-5.3	-7.4 - -3.2	**<0.001**
%Ki67+Foxp3+	6 (3-12)	15 (9-19)	27	-6.2	-9.4 – 3.0	**<0.001**

Supplementary Table 7. Changes in TILs between Day 15 and baseline according HR status, PAM50 subtype and achievement of pCR

	N (pairs)	mean difference	95% CI lower limit	95% CI upper limit	p-value
HR-positive	70	2.1%	-0.7%	4.8%	0.199
HR-positive and pCR	11	9.8%	-2.9%	22.5%	0.141
HR-positive and RD	59	0.6%	-1.7%	3.0%	0.570
HR-negative	61	12.5%	5.5%	19.6%	**<0.001**
HR-negative and pCR	23	21.0%	6.0%	36.1%	**0.014**
HR-negative and RD	38	7.3%	0.5%	14.2%	**0.029**
HER2-enriched	85	8.8%	3.5%	14.0%	**0.001**
HER2-enriched and pCR	30	15.8%	4.1%	27.5%	**0.013**
HER2-enriched and RD	55	4.9%	0.1%	10.0%	**0.038**
Non-HER2-enriched	46	3.5%	-0.3%	7.4%	0.120
Non-HER2-enriched and pCR	4	29.5%	-10.8%	69.8%	0.181
Non-HER2-enriched and RD	42	1.1%	-1.4%	3.5%	0.488

Supplementary Table 8. Distribution of tumors according to TILs at baseline, day 15 and surgery in patients not achieving pCR (N=105 patients)

**Category according to TIL levels**	**Baseline**	**Day 15**	**Surgery**
	**N (%)**	**N (%)**	**N (%)**
TILs <5%	13 (12.5%)	6 (6.1%)	10 (10.3%)
TILs 5-9%	39 (37.5%)	28 (28.6%)	37 (38.1%)
TILs 10-19%	24 (23.1%)	25 (25.5%)	22 (22.7%)
TILs 20-39%	17 (16.3%)	25 (25.5%)	16 (16.5%)
TILs ≥40%	11 (10.6%)	14 (14.3%)	12 (12.4%)
Not evaluable	N=1	N=7	N=8

Percentages calculated on the total number of evaluable samples

Supplementary Table 9. Distribution of tumors according to TILs at baseline, day 15 and surgery in patients achieving pCR (N=46 patients)

Category according to TIL levels	Baseline	Day 15	Surgery
	N (%)	N (%)	N (%)
TILs <5%	6 (13.6%)	2 (5.5%)	13(32.5%)
TILs 5-9%	12 (27.3%)	5 (13.9%)	13 (32.5%)
TILs 10-19%	5 (11.4%)	4 (11.1%)	6 (15.0%)
TILs 20-39%	12 (27.3%)	7 (19.4%)	2 (5.0%)
TILs ≥40%	9 (20.5%)	18 (50.0%)	6 (15.0%)
Not evaluable	N=2	N=10	N=6

Percentages calculated on the total number of evaluable samples

Supplementary Table 10. Changes in TILs between surgery and Day 15 according to HR status, PAM50 subtype and achievement of pathological complete response (pCR) versus residual disease (RD)

	N (pairs)	mean difference	95% CI	p-value
HR-positive				
HR-positive and pCR	11	-11.8%	-22.4 to -1.3	**0.036**
HR-positive and RD	56	+1.2%	-2.8 to +5.1	0.736
HR-negative				
HR-negative and pCR	22	-26.3%	-43.5 to -9.2	**0.011**
HR-negative and RD	35	-4.1%	-9.9 to +1.7	0.196
HER2-enriched				
HER2-enriched and pCR	29	-19.5%	-32.7 to -6.3	**0.008**
HER2-enriched and RD	50	+3.2%	-8.2 to +1.8	0.165
Non-HER2-enriched				
Non-HER2-enriched and pCR	4	-36.0%	-62.2 to -9.8	**0.022**
Non-HER2-enriched and RD	41	+2.0%	-2.0 to +5.9	0.353

Supplementary Table 11. Proportion of patients with different tumor-infiltrating lymphocytes (TILs) dynamics across the three different timepoints assessed in the PAMELA trial (N=122 patients with TILs data from all three timepoints)

From Baseline to Day 15			From Day 15 to Surgery		
	N	%		N	%
Increasing TILs	51	41.8	Increasing TILs	4	7.8
			Unchanged TILs	15	29.4
			Decreasing TILs	32	62.7
Unchanged TILs	46	37.7	Increasing TILs	16	34.8
			Unchanged TILs	16	34.8
			Decreasing TILs	14	30.4
Decreasing TILs	25	20.5	Increasing TILs	6	24
			Unchanged TILs	13	52
			Decreasing TILs	6	24

Supplementary Table 12. Changes in TILs between surgery and baseline in patients achieving pCR and not achieving pCR according to HR status and PAM50 subtype

	N (pairs)	mean difference	95% CI - lower limit	95% CI - upper limit	p-value
**Patients achieving pCR**	**39**	**-8.3%**	**-16.0%**	**-0.6%**	**0.02**
HR-status					
HR-positive	14	-1.6%	10.1%	-13.2%	0.456
HR-negative	25	-12.1%	-22.5%	1.7%	**0.023**
PAM50 subtype					
HER2-enriched	35	-8.5%	-17.1%	0.1%	**0.035**
Non-HER2-enriched	4	-6.5%	-22.3%	9.3%	0.414
**Patients with residual disease**	**96**	**2.3%**	**-1.2%**	**5.8%**	0.069
HR-status					
HR-positive	60	2.1%	-1.6%	5.7%	0.286
HR-negative	36	2.6%	-4.7%	10.0%	0.107
PAM50 subtype					
HER2-enriched	54	1.3%	-4.4%	7.0%	0.404
Non-HER2-enriched	42	3.6%	0.1%	7.0%	0.059

Supplementary Table 13. Complete lists of genes evaluated using the nCounter platform (plus five housekeeping genes: ACTB, MRPL19, PSMC4, RPLP0, SF3A1)

A1CF
AARS
ABAT
ABCB1
ABCC3
ABCC8
ACOT4
ACTL8
ACTR3B
ADM
ADRA2A
ADRA2C
AFF3
AGR2
AGR3
AHCYL1
AKT1
AKT3
ALDH1A1
ANGPTL4
ANLN
ANXA1
ANXA8L2
APC
APH1B
AR
ARAF
AREG
ASF1A
ATAD2
ATAD3A
ATM
ATR
AURKA
AVEN
AVL9
AXL
AZGP1
BAG1
BCL11A
BCL2
BCL2A1
BDNF
BIRC5
BLM
BLVRA
BMI1
BOP1
BRAF

BRCA1
BRCA2
BTG2
BTG3
BUB1
BYSL
C11orf30
C12orf11
C16orf45
C16orf61
C1orf106
C1orf21
C21orf45
C4orf32
C4orf34
C8orf30A
C8orf33
CA12
CABP7
CAMK2N1
CAND1
CAPN13
CAPN6
CAV1
CBX7
CCDC86
CCNA2
CCNB1
CCND1
CCND2
CCND3
CCNE1
CD19
CD24
CD274
CD3G
CD4
CD44
CD68
CD84
CD86
CD8A
CDA
CDC123
CDC20
CDC25B
CDC25C
CDC45L
CDC6
CDCA1
CDCA5

CDCA7
CDCA7L
CDCA8
CDH1
CDH3
CDK1
CDK4
CDKN1A
CDKN1B
CDKN2A
CDKN2B
CDKN2C
CDKN2D
CDKN3
CDT1
CDYL
CEACAM6
CELSR1
CENPA
CENPF
CENPI
CENPN
CEP55
CFLAR
CHEK1
CHEK2
CHPF
CHST11
CHUK
CITED4
CKS1B
CKS2
CLDN3
CLDN4
CLDN7
CLMN
COG8
COX4NB
COX6C
COX7B
CRIM1
CRYAB
CSDA
CTGF
CTNNB1
CTPS
CTSL1
CTSL2
CXCL1
CXCL14
CXCR1

CXCR2
CXXC5
CYB5B
CYBRD1
CYCS
CYR61
DDB2
DDIT4
DDR1
DEGS2
DLGAP5
DNAJC12
DNALI1
DSP
E2F1
ECE2
EGFR
EIF2S2
ELOVL5
ELSPBP1
EMP3
EPCAM
EPN3
EPSTI1
ERBB2
ERBB3
ERBB4
ERCC1
ESR1
ESRP1
EVI2A
EXO1
EZH2
F11R
F3
FABP4
FABP5
FAM171A1
FAM174B
FAM198B
FAM38A
FAM54A
FANCA
FANK1
FAP
FBN1
FBP1
FBXL6
FGFR1
FGFR2
FGFR4

FIGF
FLVCR2
FNBP1
FOXA1
FOXC1
FOXM1
FREQ
FZD6
FZD7
GABPB1
GAL
GALNT7
GARS
GATA3
GGH
GINS2
GLRB
GNG11
GOLT1A
GPR160
GPR172A
GPR89A
GPSM2
GRB7
GREM1
GRHL1
GRHL2
GSTM1
GSTM3
GSTM4
GSTP1
GTPBP4
GUSB
H19
HEXIM1
HIF1A
HJURP
HMGA1
HN1
HRAS
HSPA14
HSPD1
hypothetical.LOC389332
hypothetical.LOC400043
hypothetical.protein.LOC642077
hypothetical.protein.LOC647456
hypothetical.protein.MGC18216
ID4
IDH2
IDO1
IFT74

IGBP1
IGF1
IGF2R
IGFBP2
IKBKB
IKBKE
IL1B
IL6
IL6R
IL6ST
IL8
INHBA
INPP4B
INSIG1
IRX3
ITCH
ITGA6
ITGB1
JUP
KCNJ15
KCTD1
KDM4B
KDR
KIAA0040
KIAA1324
KIAA1370
KIF13B
KIF20A
KIF23
KIF2C
KIF4A
KIFC1
KIT
KLF4
KLHL7
KLHL9
KNTC2
KPNA1
KRAS
KRT14
KRT16
KRT17
KRT18
KRT19
KRT23
KRT5
KRT6A
KRT6B
KRT6C
KRT8
KRTAP1.1

LAG3
LAMA3
LAMC2
LEPRE1
LHFP
LRIG1
LRP8
LRRC2
LSR
LTBP2
MAD2L1
MAGEA1
MAGOHB
MAP2K1
MAP2K4
MAP7D3
MAPT
MCM2
MCM3
MDM2
ME1
MED21
MELK
MET
MIA
MIEN1
MKi67
MKRN2
MLKL
MLPH
MME
MMP11
MPP1
MRPS17
MRPS35
MS4A1
MSH2
MTHFD1L
MTOR
MUC1
MUC5B
MYB
MYBL2
MYC
MYO5C
NACC2
NAT1
NCAPH2
NDRG1
NDUFAF4
NEK2

NEO1
NF1
NFIA
NFIB
NFKB1
NFKBIA
NFKBIB
NFKBIE
NLN
NOP56
NOTCH1
NOTCH2
NOTCH3
NPEPPS
NPM2
NQO1
NR4A3
NRAS
NT5E
NTN4
NUDCD1
NUDT1
NUP88
NUP93
OCLN
OGFRL1
OGN
ORC6L
P4HTM
PARP1
PCDH8
PCNA
PDCD1
PDGFRA
PDSS1
PDXK
PEX11G
PGAM5
PGR
PHGDH
PID1
PIK3CA
PIK3R1
PIP
PIR
PITX1
PLA1A
PLOD1
PNO1
PNP
POLD1

PPFIBP1
PRAME
PRC1
PREP
PROM1
PSMA7
PSMD14
PSPH
PSPHL
PTDSS1
PTEN
PTGER4
PTGS2
PTTG1
PUF60
PUM1
PVRL3
PYROXD1
RAB25
RAB35
RACGAP1
RAD17
RAD50
RAD51
RAD51C
RAD51L1
RAF1
RAI2
RANBP1
RARA
RB1
RBBP8
RECK
RECQL
REEP6
RELA
RELB
RERG
RFC4
RGS22
RHBG
RINT1
RNF103
RRAGD
RRM2
RRP15
S100A11
S100A14
S100A8
S100A9
SAPS1

SCGB2A2
SCUBE2
SEH1L
SEMA3C
SERPINA3
SETBP1
SFRP1
SH2B3
SHC1
similar.to.Keratin.type.II.cytoskeletal.8..Cytokeratin.8...CK.8...Keraton.8...K8.
SLC16A3
SLC25A19
SLC39A6
SLC40A1
SLC5A6
SLC7A6
SLC9A3
SLC9A3R1
SMO
SNAI1
SNRPA1
SNRPD1
SPAG5
SPATA7
SPDEF
SPINT1
SPINT2
SQLE
SRC
ST18
STAT1
STAT3
STC2
STK11
STK38L
STMN1
STRAP
SUV39H2
TACC3
TAP1
TCEAL1
TCF7L1
TFAM
TFF1
TFF3
TFRC
TGFBR2
TGFBR3
THBS1
THY1
TIMM17A

TIMM8A
TK1
TM7SF3
TMCC2
TMEM125
TMEM139
TMEM158
TMEM208
TMEM25
TMEM45B
TNFRSF11A
TNFSF11
TOM1L1
TOMM40
TOP2A
TOR1A
TP53
TP53BP2
TP63
TRIM29
TRIP13
TSHZ1
TSPAN13
TTK
TUBA4A
TUBB6
TWIST1
TWIST2
TYMP
TYMS
UBE2C
UBE2T
UCHL1
UIMC1
USP10
VAMP8
VAV3
VEGFA
VIM
WDR12
WDR4
WIPF2
XBP1
YBX1
ZEB1
ZEB2
ZNF217

Supplementary Table 14. Genes up-regulated in association with TIL levels at quantitative SAM analysis (all timepoint combined, samples N=413) with False Discovery Rate <1%

Gene ID	Score(d)	q-value(%)
MS4A1	8.903867	0
CD19	8.57364	0
LAG3	8.441398	0
CD3G	8.390272	0
CD8A	8.129773	0
PDCD1	7.589843	0
TAP1	6.742996	0
CD274	6.401272	0
IDO1	5.978856	0
STAT1	5.965263	0
IL6R	5.559205	0
CD4	5.446276	0
CD86	5.243833	0
MLKL	5.084844	0
EPSTI1	4.915366	0
IKBKE	4.900681	0
CHST11	4.742928	0
CD84	4.517602	0
NFKBIE	4.434747	0
BCL2A1	4.370058	0
TYMP	4.32247	0
EVI2A	4.302834	0
RELB	3.945947	0
TUBA4A	3.797016	0
CD68	3.674341	0
FLVCR2	3.513351	0
CDC25B	3.394281	0
CFLAR	3.336673	0
MPP1	3.166783	0
EMP3	3.165354	0
PTGER4	3.153685	0
SAPS1	2.744665	0
BLM	2.677925	0
CD44	2.631944	0
BTG2	2.51612	0.205387
ABCB1	2.319839	0.861731

Supplementary Table 15. Genes up-regulated in association with TIL levels at quantitative SAM analysis at baseline (samples N=148) with False Discovery Rate <1%

Gene ID	Score(d)	q-value(%)
CD8A	6.22832	0
CD3G	5.957181	0
LAG3	5.692738	0
MS4A1	5.661252	0
CD19	5.569917	0
PDCD1	5.569657	0
IDO1	5.547896	0
CD274	5.272603	0
STAT1	5.11594	0
TAP1	5.085964	0
EPSTI1	4.766666	0
IL6R	4.496497	0
NFKBIE	4.293866	0
MLKL	4.10485	0
BCL2A1	4.093467	0
CD4	4.058705	0
CFLAR	3.942835	0
RELB	3.631598	0
CHST11	3.566292	0
EVI2A	3.551481	0
CD86	3.326293	0
IKBKE	3.058797	0
CD84	2.997707	0
EMP3	2.97741	0
CCND2	2.928956	0
PLA1A	2.916057	0
TUBA4A	2.785798	0
TYMP	2.758991	0
PTGER4	2.746747	0
ABCB1	2.698752	0
BTG2	2.426338	0.675158
CDC25B	2.419813	0.675158

Supplementary Table 16. Genes up-regulated in association with TIL levels at quantitative SAM analysis at Day 15 (samples N=133) with False Discovery Rate <1%

Gene ID	Score(d)	q-value(%)
MS4A1	4.9853006	0
PDCD1	4.667656	0
CD19	4.6125682	0
CD8A	4.4783514	0
LAG3	4.4566158	0
CD3G	4.4164114	0
CD274	3.2353967	0
IL6R	3.1299524	0
TAP1	2.9735575	0
STAT1	2.8611134	0
CD4	2.8579478	0
EPSTI1	2.769441	0
BCL2A1	2.7582879	0
CD86	2.6210126	0
CHST11	2.6114742	0
IDO1	2.5656666	0
IKBKE	2.3961171	0
MLKL	2.3618481	0
EVI2A	2.3408257	0
TUBA4A	2.2860373	0
CD84	2.263514	0
FLVCR2	2.2223425	0
CD68	2.1331608	0.241761
BTG2	2.0955087	0.241761
NFKBIE	2.0763461	0.241761
BLM	2.0478201	0.449391
CDC25B	1.9141768	0.834933
TYMP	1.8918392	0.834933
MPP1	1.8752434	0.834933

Supplementary Table 17. Genes up-regulated in association with TIL levels at quantitative SAM analysis at Surgery (samples N=132) with False Discovery Rate <1%

Gene ID	Score(d)	q-value(%)
MS4A1	3.959441	0
CD19	3.555171	0
CD8A	2.69107	0
CD3G	2.428863	0
LAG3	2.422838	0
TAP1	2.400816	0
TYMP	2.334461	0
HMGA1	2.270879	0
BLM	2.169032	0
PRAME	2.01589	0
IDO1	2.012051	0
MKi67	1.948502	0
CEP55	1.890432	0
FOXM1	1.825431	0
PDCD1	1.783179	0
CDC6	1.773788	0
CCNA2	1.741049	0
KRT8	1.738559	0
IKBKE	1.726431	0
KIFC1	1.718271	0
HJURP	1.715825	0
TOP2A	1.713789	0

Supplementary Table 18. NGI antibodies and protocols

ANTIBODY	VENDOR	REFERENCE	ANTIGEN RETRIEVAL	PRIMARY ANTIBODY	PRIMARY ANTIBODY DILUTION	SECUNDARY ANTIBODY	CHROMOGEN
FOXP3	ABCAM	AB99963	CC1 92' 95º	1h RT	1/100	HRP RB 20'	AECPLUS. 150UL
CD4 (SP35)	ROCHE	790-4423	CC2 8' 100º<br>CC1 95º 56'	52' 36º	RTU	HRP RB 16'	AECPLUS. 200UL
KI67 (MIB-1)	DAKO	M7240	CC2 8' 100º<br>CC1 72' 95º	1h 32' RT	1/100	HRP MS 20'	AECPLUS. 150UL
CD8 (SP57)	ROCHE	790-4460	CC2 8' 100º	28' 36º	RTU	HRP RB 8'	AECPLUS. 200UL
CD3 (2GV6)	ROCHE	790-4341	CC2 8' 100º<br>CC1 40' 95º	40' 36º	RTU	HRP RB 8'	AECPLUS. 200UL
AE1/AE3/PCK26	ROCHE	760-2595	CC2 8' 100º<br>CC1 40' 95º	24' 36º	RTU	HRP MS 8'	AECPLUS. 200UL

Supplementary Table 19. T-cell APP details

Magnification	20X
Classification method	Cell classification
Classification feature	CD3 channel- HDAB-DAB<br>Sensitivity: 98%<br>Size: 15um
Post processing<br>Change by area<br>Separate objects<br>Change by area<br>Change by area<br>Separate objects<br>Change<br>Change by intensity (Foxp3 channel)<br>Change by intensity (CD8 channel)<br>Change by intensity (CD4 channel)<br>Change by intensity (Ki67 channel)<br>Change by intensity (Ki67 channel)<br>Change by intensity (Ki67 channel)<br>Change by intensity (Ki67 channel)<br>Apply counting frame	 Lbl: CD3+, Max: 10un2<br>Lbl: CD3+. Object diameter 7um<br>Lbl: CD3+, Max: 15un2, Clear<br>Lbl: CD3+, Max: 30un2, label 2<br>Lbl: label 2. Object diameter 5um2<br>Lbl: label 2 to CD3+<br>Lbl: CD3; intensity: -inf -> 150, %object 0%-30%, Foxp3+<br>Lbl: CD3; intensity: -inf -> 200, %object 0%-20%, CD8+<br>Lbl: CD3; intensity: -inf -> 200, %object 0%-20%, CD4+<br>Lbl: CD3; intensity: -inf -> 175, %object 0%-30%, CD3+Ki67+<br>Lbl: Foxp3; intensity: -inf -> 200, %object 0%-20%, Foxp3+Ki67+<br>Lbl: CD8; intensity: -inf -> 175, %object 0%-30%, CD8+Ki67+<br>Lbl: CD4; intensity: -inf -> 175, %object 0%-30%, CD4+Ki67+<br>Lbl: all, replace with clear
Output	Densities of all classes:<br>Foxp3+ Ki67+<br>Foxp3+ Ki67-<br>CD3+ Ki67+<br>CD3+ Ki67-<br>Cd8+ Ki67+<br>Cd8+ Ki67-<br>Cd4+Ki67+<br>Cd4+Ki67-<br>% of proliferating immune cells:<br>% proliferating Foxp3<br>% proliferating CD3<br>% proliferating CD8<br>% proliderating CD4<br>Areas

Supplementary Figures

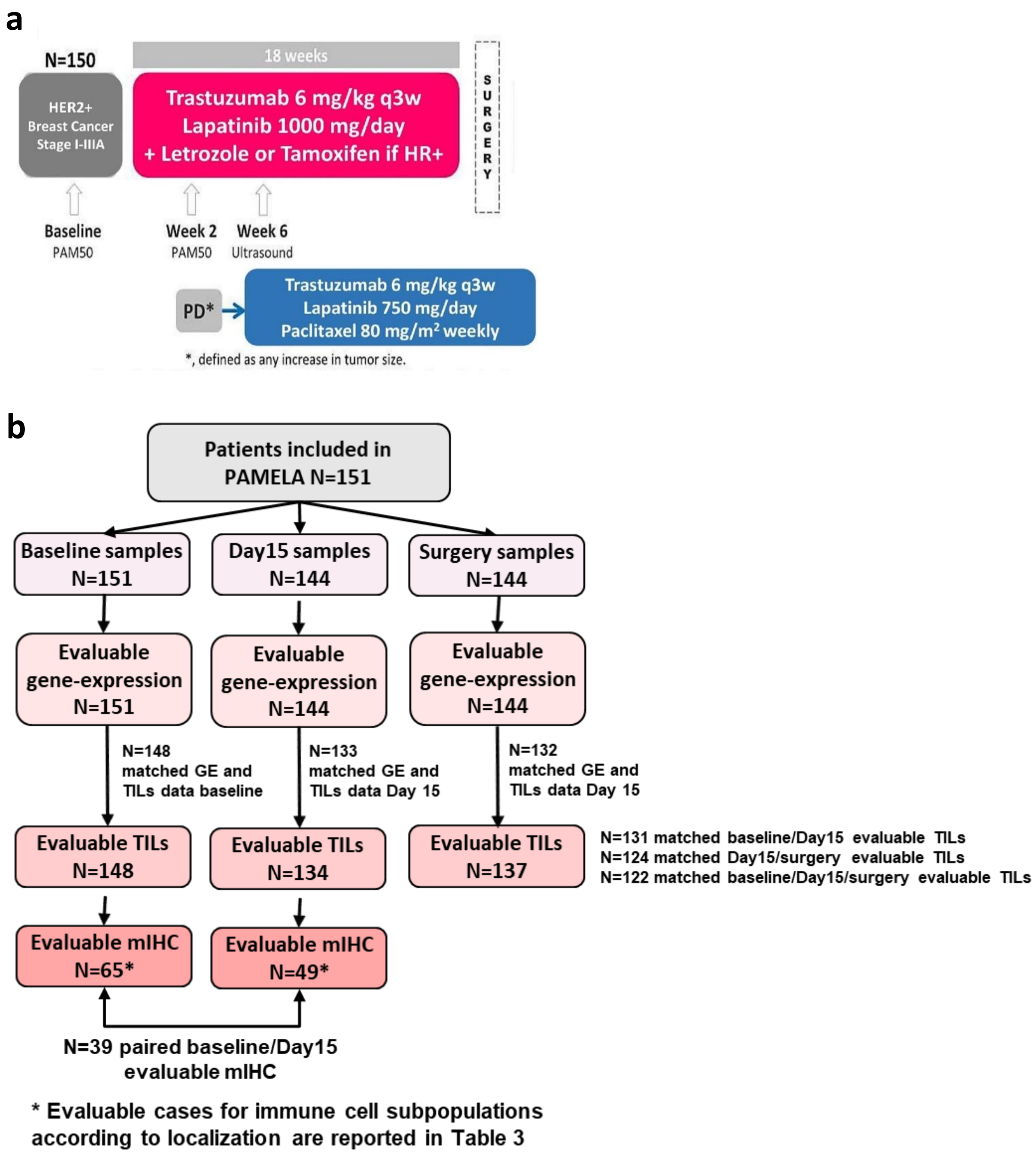

Supplementary Figure 1. PAMELA trial design (a) and REMARK diagram of biological samples evaluated in this translational trial (b)

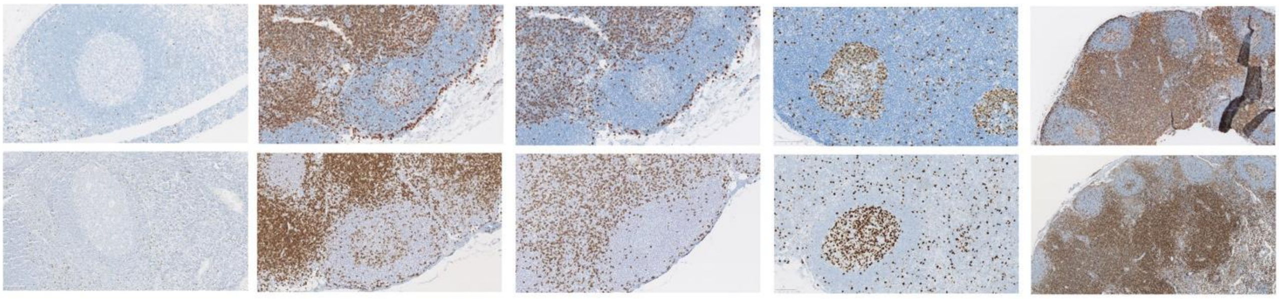

Supplementary Figure 2. Representative stainings of Foxp3, CD3, CD8, KI67 and CD4 markers (from left to right) performed with NGI technology in the upper line and with standard DAB staining in the bottom line. Foxp3, CD3, CD8 and KI67 images at 20X and CD4 images at 10X.

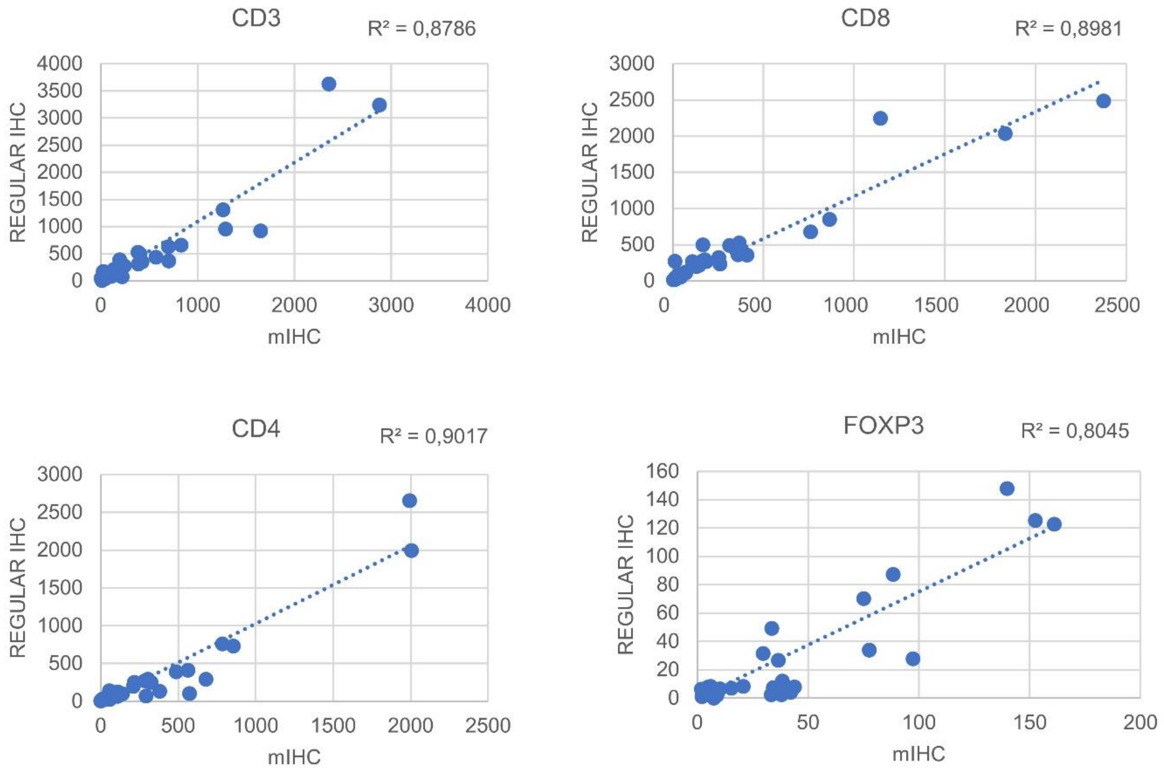

Supplementary Figure 3. Correlations of densities of different biomarkers performed with NGI technology (mIHC) and with regular IHC protocols using DAB in a subset of 30 breast cancer tumors on a tissue microarray.

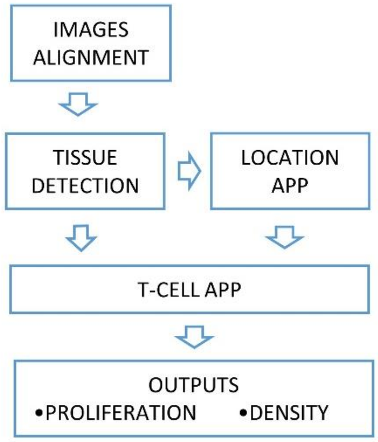

Supplementary Figure 4. Analytical pipeline of image analysis. First, obtained images are aligned. Then, an automatic tissue recognition is performed and after that the t-cell density APP is run to obtain global results. In order to obtain results separately for each of the areas, the location APP is run before the t-cell density APP.

Baseline:

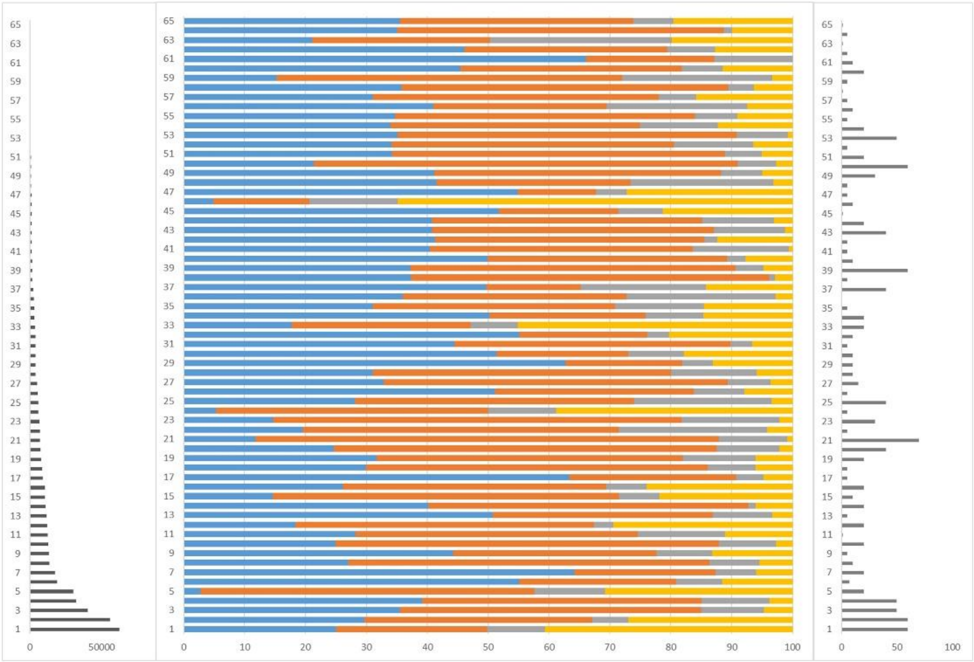

Day 15:

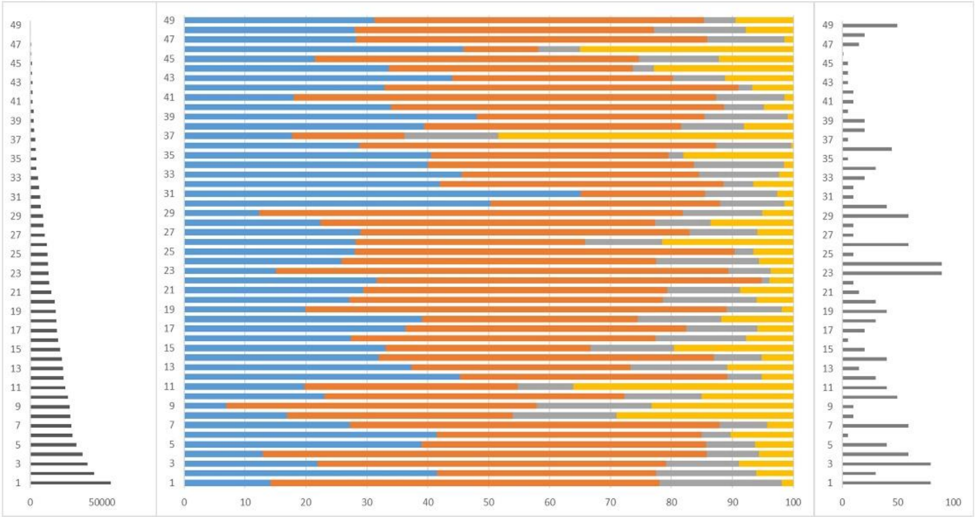

Supplementary Figure 5. CD3+ densities, proportions of CD8⁺ (blue), Foxp3⁺ (orange), CD4⁺ (gray) and CD3⁺only (yellow) cells and stromal tumor infiltrating lymphocytes (TILs) separated by baseline and day15 samples.

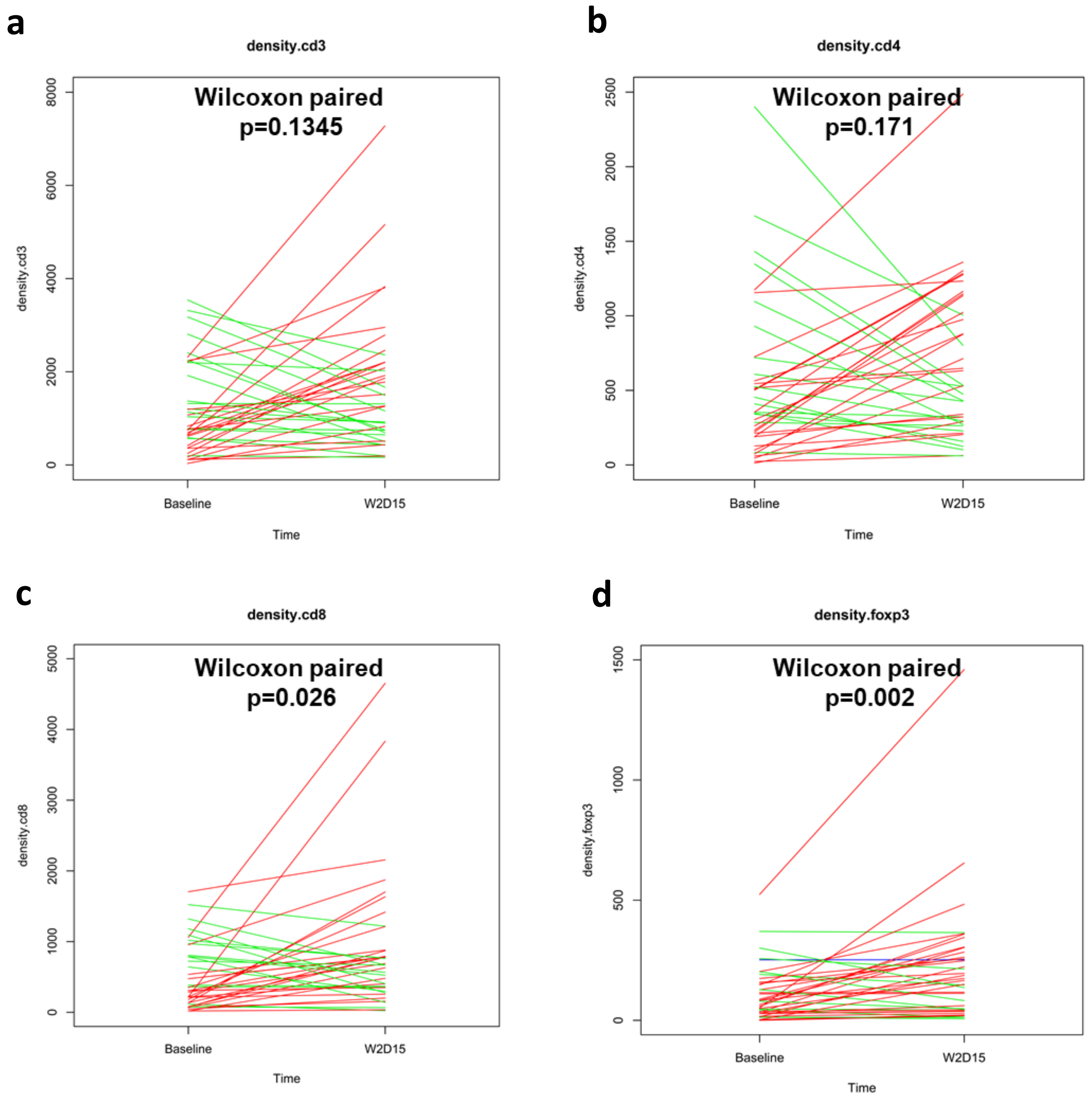

Supplementary Figure 6. Two-point graphs showing individual patient immune cell density data from baseline to Day 15 for the 39 patients with paired samples: CD3 (a); CD4 (b); CD8 (c); Foxp3 (d).

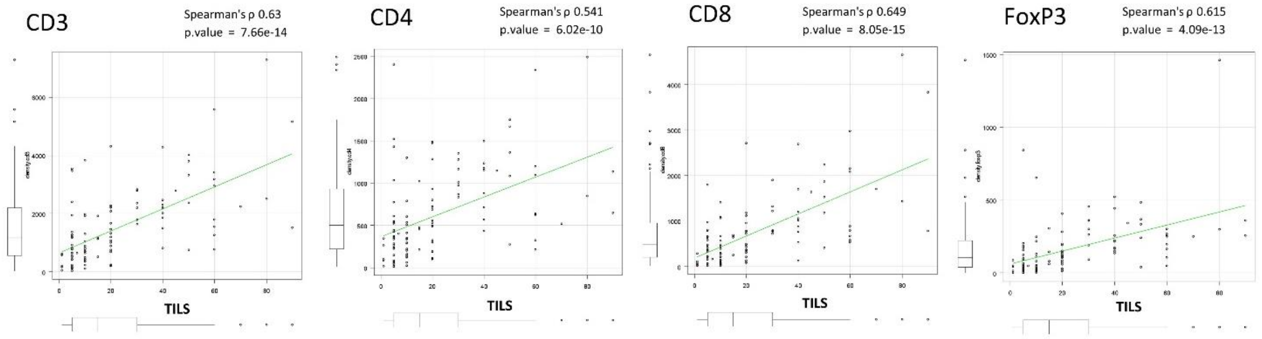

Supplementary Figure 7. Scatter plots showing the correlation between immune cell densities (CD3+, CD4+, CD8+ and Foxp3+) and TILs. Box-plot of immune cell densities and TILs are shown next to the y and x axis, respectively.

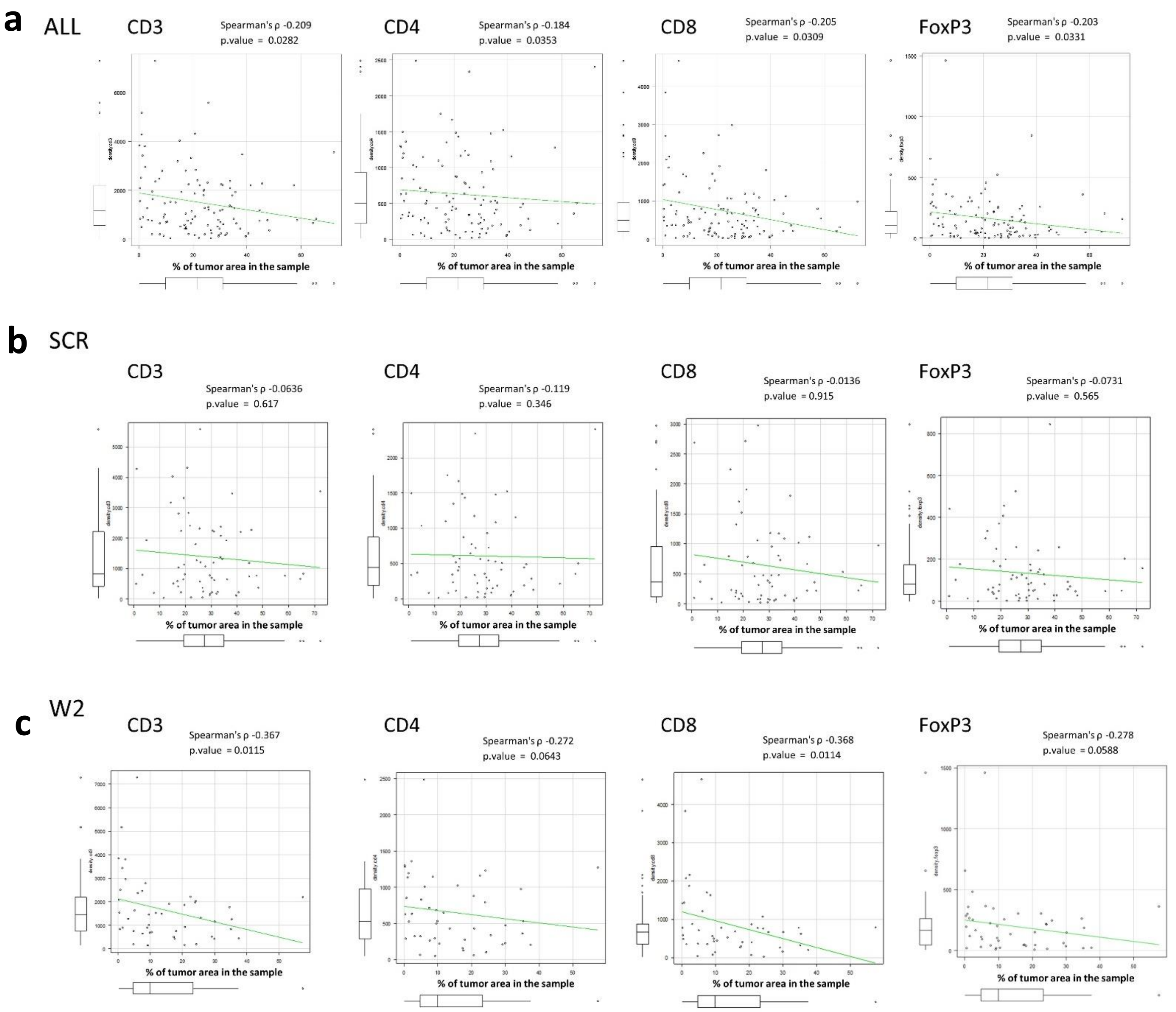

Supplementary Figure 8. Scatter plots showing the correlation between immune cell densities (CD3+, CD4+, CD8+ and Foxp3+) and percentage of tumor area in the sample. Box-plot of immune cell densities and percentage of tumor area in the sample are shown next to the y and x axis, respectively. a, all samples, b, baseline samples and c, Day 15 samples.

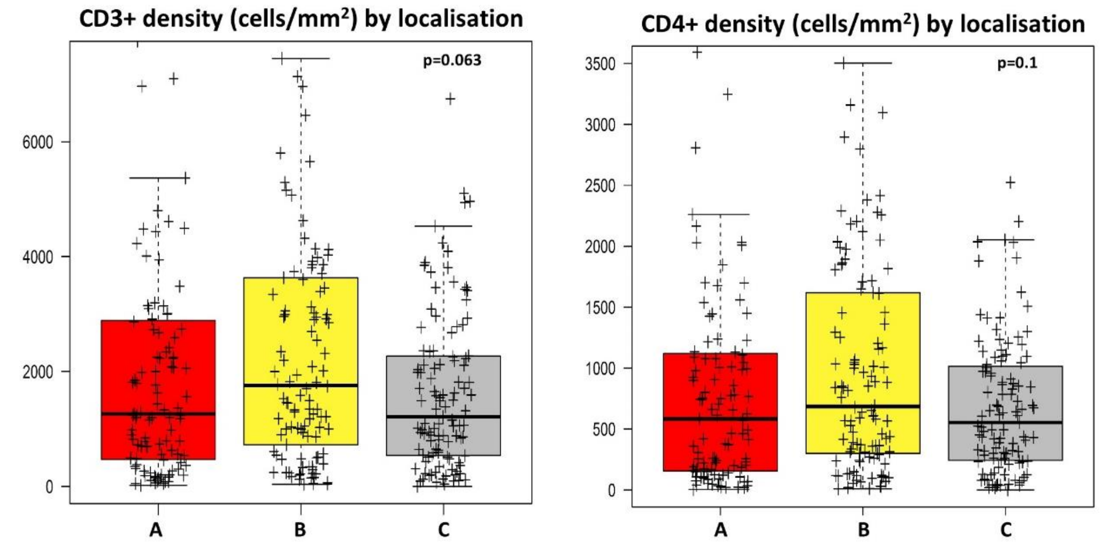

Supplementary Figure 9. Box-plots of immune cells densities (CD3+and CD4+) according to spatial location. Boxplot legend: centre line: median; bounds of box: interquartile range (IQR); whiskers: highest and lowest value excluding outliers (Q3+1.5*IQR to Q1-1.5*IQR); markers beyond the whiskers: potential outliers.

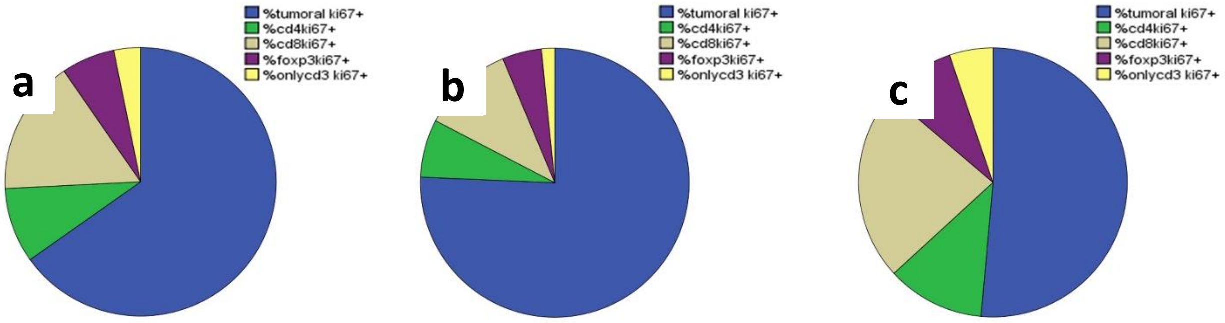

Supplementary Figure 10. Pie chart showing the proportion of proliferating cells over the total proliferating cells in the tumor samples. Tumoral cells in blue, CD4 in green, CD8 in gray, Foxp3 in purple and CD3 only in yellow. a for all the samples, b for baseline samples and c for Day15 samples.

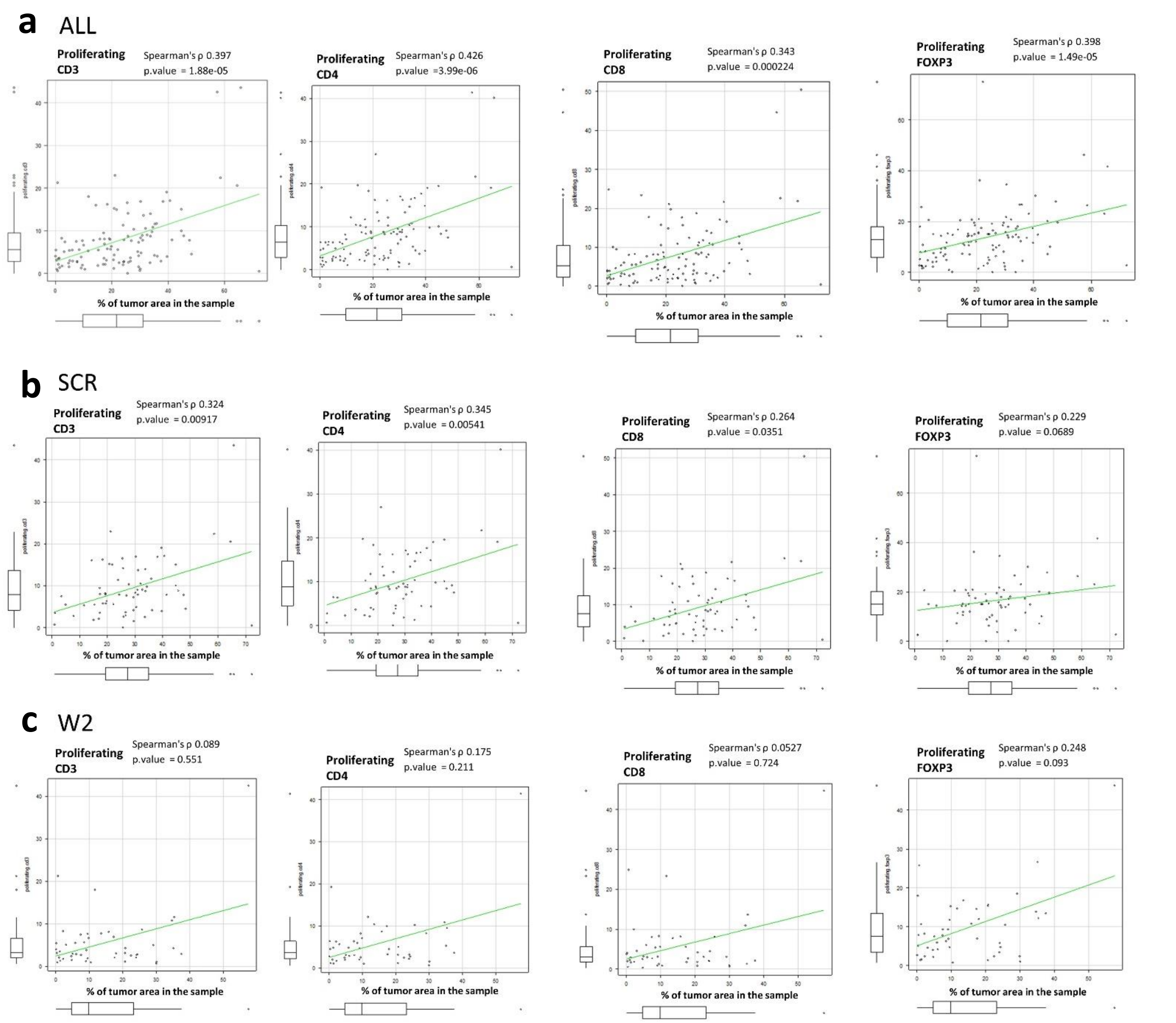

Supplementary Figure 11. Scattered plots of the proportion of proliferating cells (CD3+, CD4+, CD8+ and Foxp3+) and tumoral areas. Box-plot of the proportion of proliferating cells and tumoral areas are shown next to the y and x axis, respectively. a for all the samples, b for baseline samples and c for Day 15 samples.

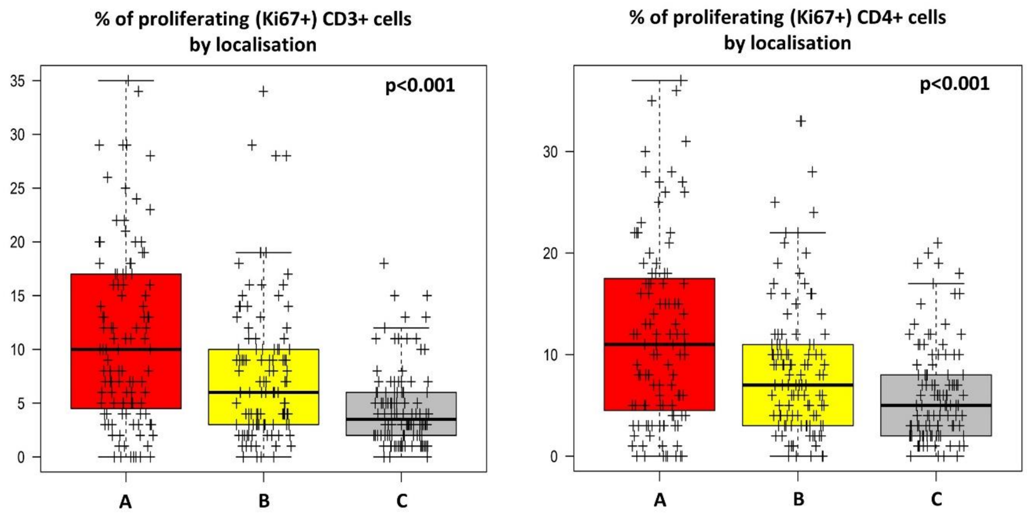

Supplementary Figure 12. Box-plots of the proportion of proliferating immune cells (CD3+ and CD4+) according to spatial location. Boxplot legend: centre line: median; bounds of box: interquartile range (IQR); whiskers: highest and lowest value excluding outliers (Q3+1.5*IQR to Q1-1.5*IQR); markers beyond the whiskers: potential outliers.

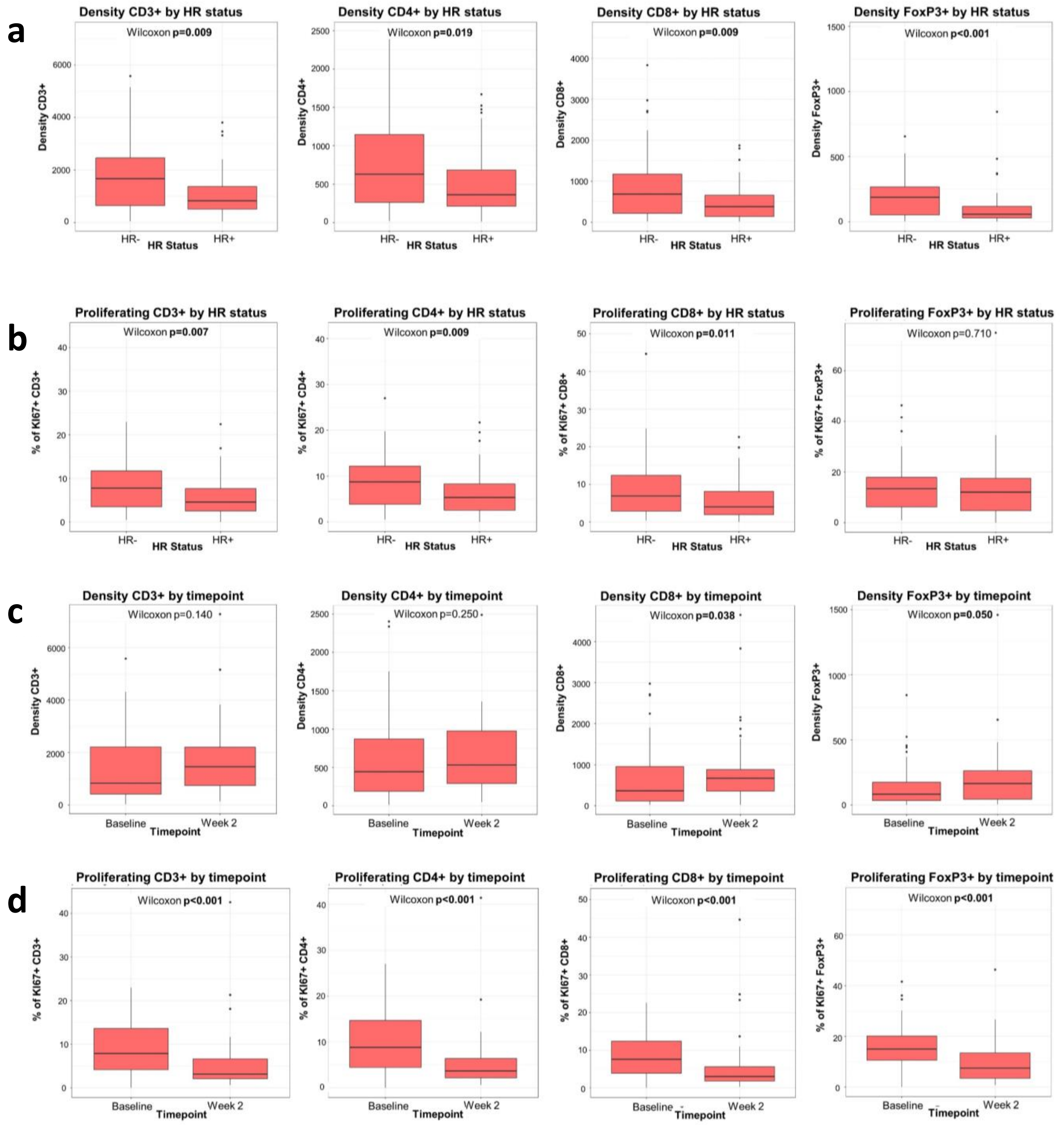

Supplementary Figure 13. Box-plots of immune cell densities (CD3+, CD4+, CD8+ and Foxp3+) and percentage of proliferating immune cells (CD3+, CD4+, CD8+ and Foxp3+) according to HR status (a and b, respectively) and according to timepoint (c and d, respectively). Boxplot legend: centre line: median; bounds of box: interquartile range (IQR); whiskers: highest and lowest value excluding outliers (Q3+1.5*IQR to Q1-1.5*IQR); markers beyond the whiskers: potential outliers.

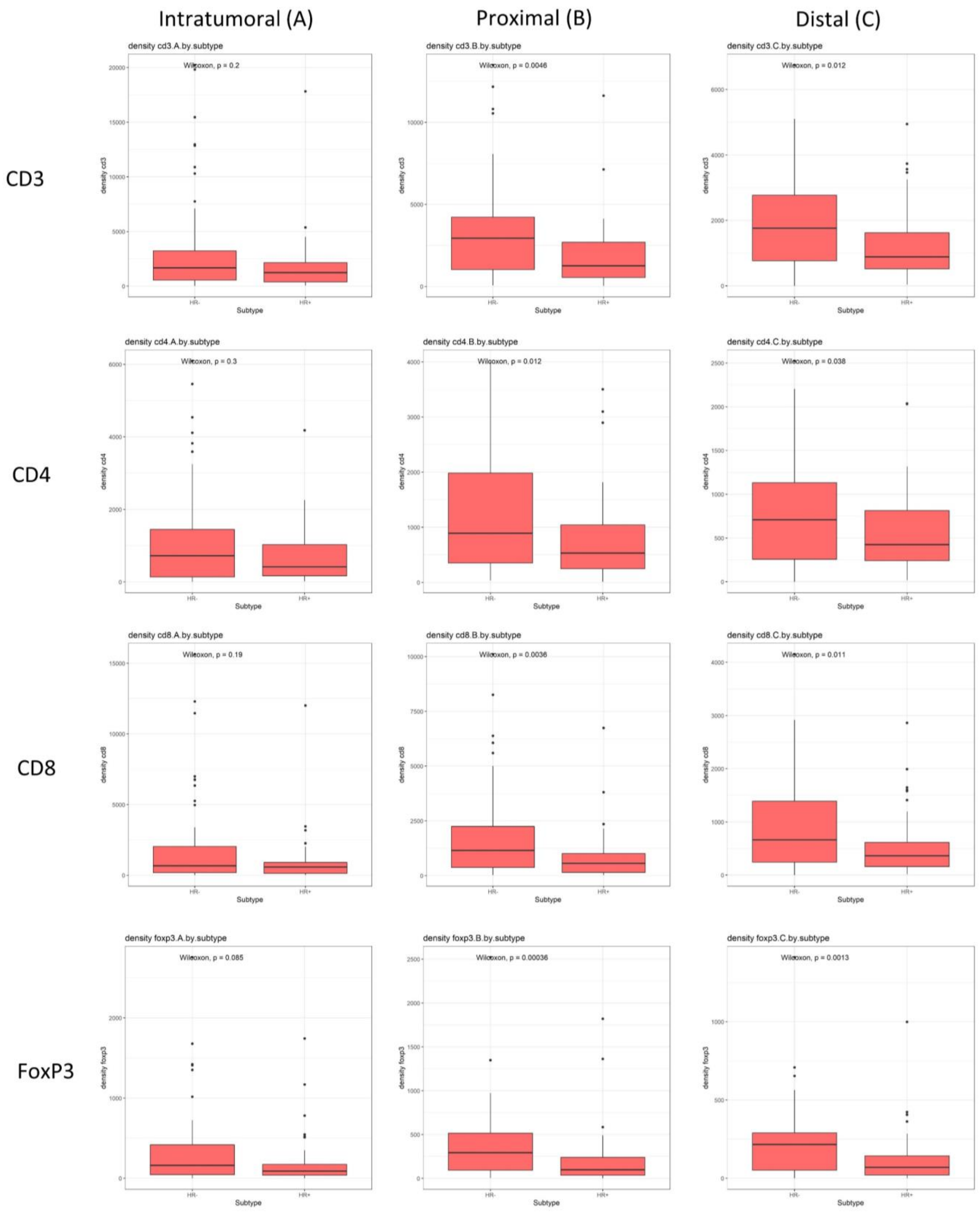

Supplementary Figure 14. Box-plots of immune cell densities (CD3+, CD4+, CD8+ and Foxp3+) by HR status in intratumoral (A), proximal (B) and distal (C) locations. Boxplot legend: centre line: median; bounds of box: interquartile range (IQR); whiskers: highest and lowest value excluding outliers (Q3+1.5*IQR to Q1-1.5*IQR); markers beyond the whiskers: potential outliers.

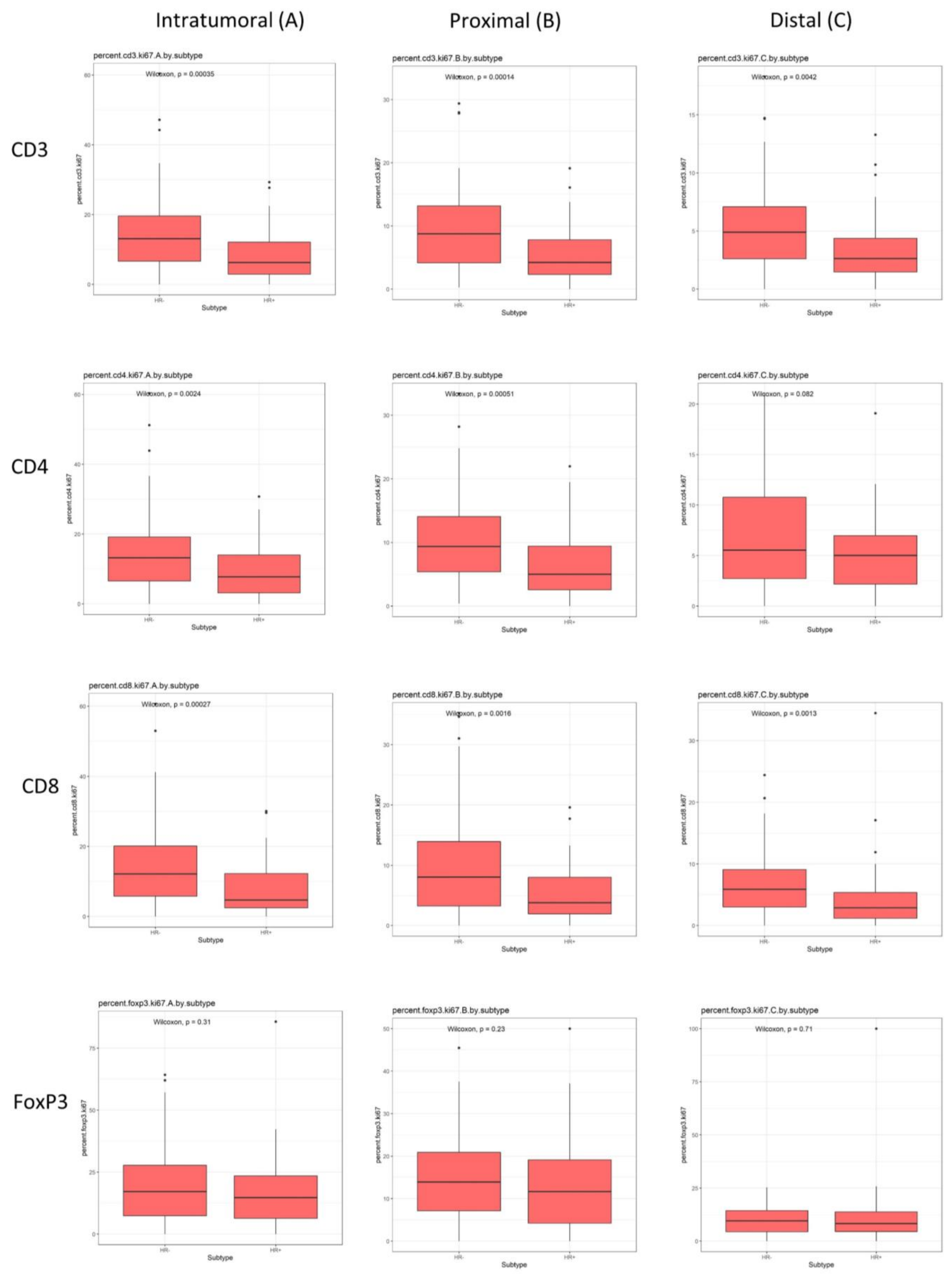

Supplementary Figure 15. Box-plots of percentage of proliferating immune cells (CD3+, CD4+, CD8+ and Foxp3+) by HR status in intratumoral (A), proximal (B) and distal (C) locations. Boxplot legend: centre line: median; bounds of box: interquartile range (IQR); whiskers: highest and lowest value excluding outliers (Q3+1.5*IQR to Q1-1.5*IQR); markers beyond the whiskers: potential outliers.

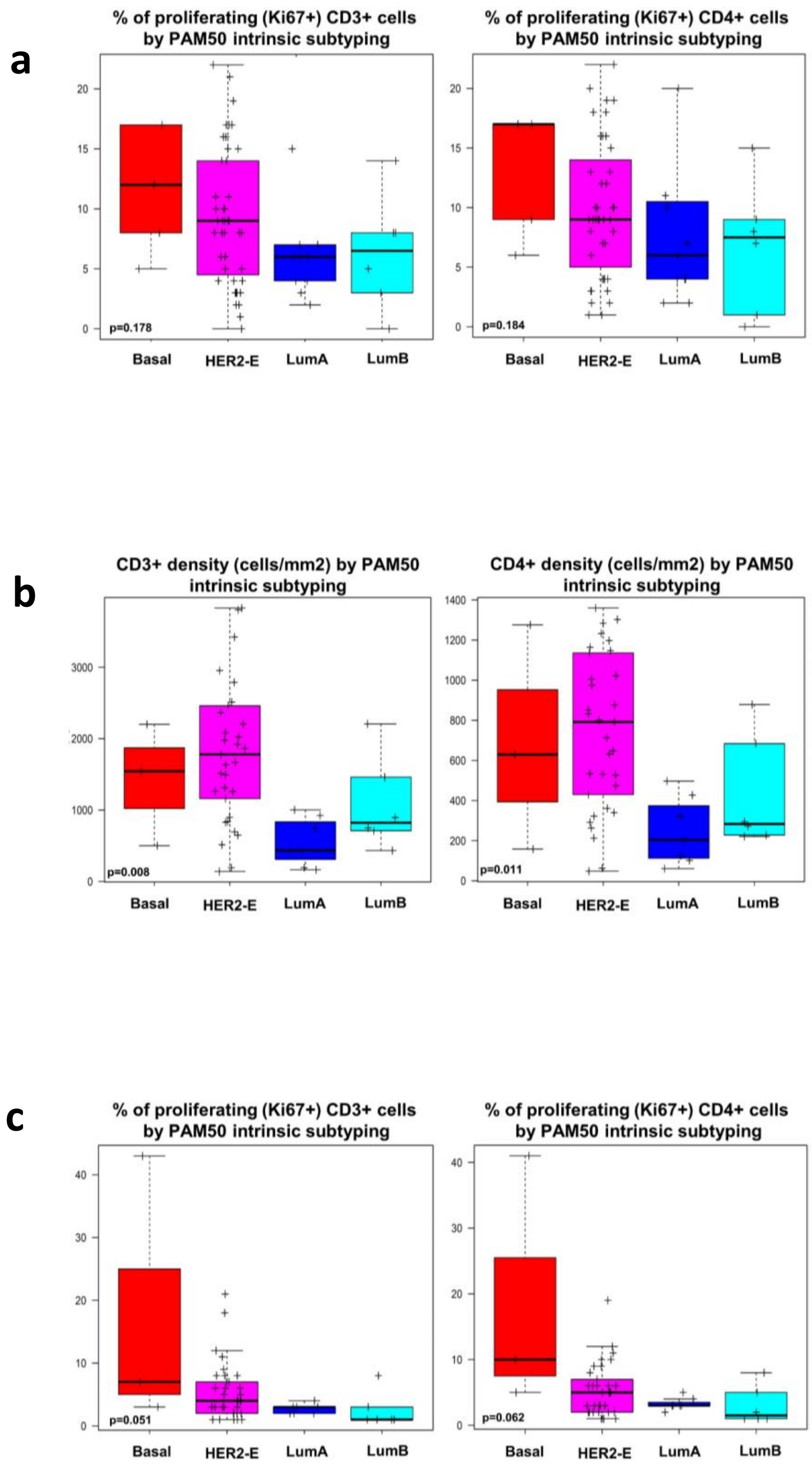

Supplementary Figure 16. Immune cell density according to intrinsic subtype

Supplementary Figure 16a. Box-plots of the proportion of proliferating immune cells ($CD3^+$, $CD4^+$) at baseline according to baseline intrinsic subtyping.

Supplementary Figure 16b. Box-plots of immune cells densities ($CD3^+$, $CD4^+$) at day 15 according to baseline intrinsic subtyping.

Supplementary Figure 16c. Box-plots of the proportion of proliferating immune cells ($CD3^+$, $CD4^+$) at day 15 according to baseline intrinsic subtyping.

Boxplot legend: centre line: median; bounds of box: interquartile range (IQR); whiskers: highest and lowest value excluding outliers (Q3+1.5*IQR to Q1-1.5*IQR); markers beyond the whiskers: potential outliers.

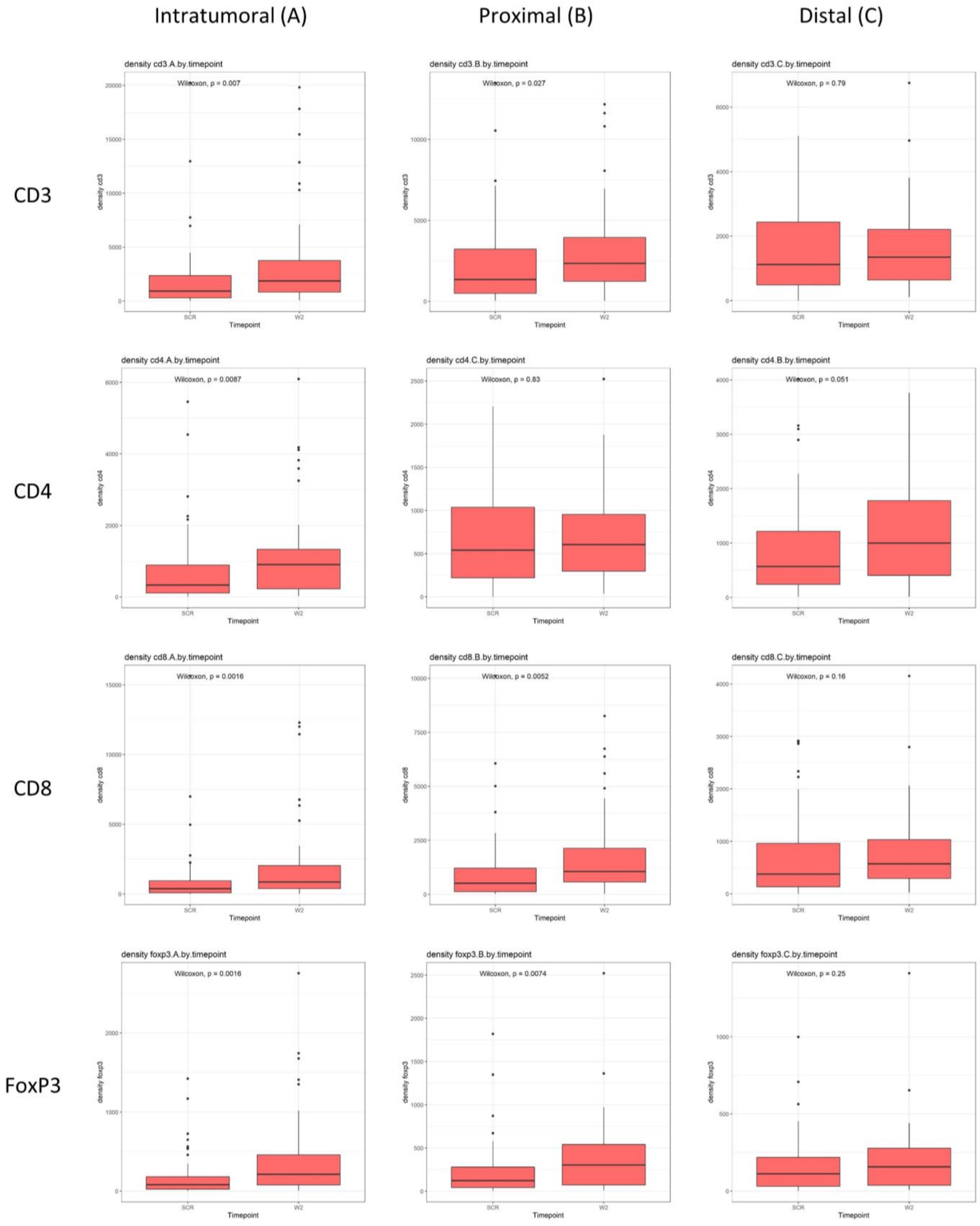

Supplementary Figure 17. Box-plots of immune cell densities (CD3+, CD4+, CD8+ and Foxp3+) by timepoint in intratumoral (A), proximal (B) and distal (C) locations. Boxplot legend: centre line: median; bounds of box: interquartile range (IQR); whiskers: highest and lowest value excluding outliers (Q3+1.5*IQR to Q1-1.5*IQR); markers beyond the whiskers: potential outliers.

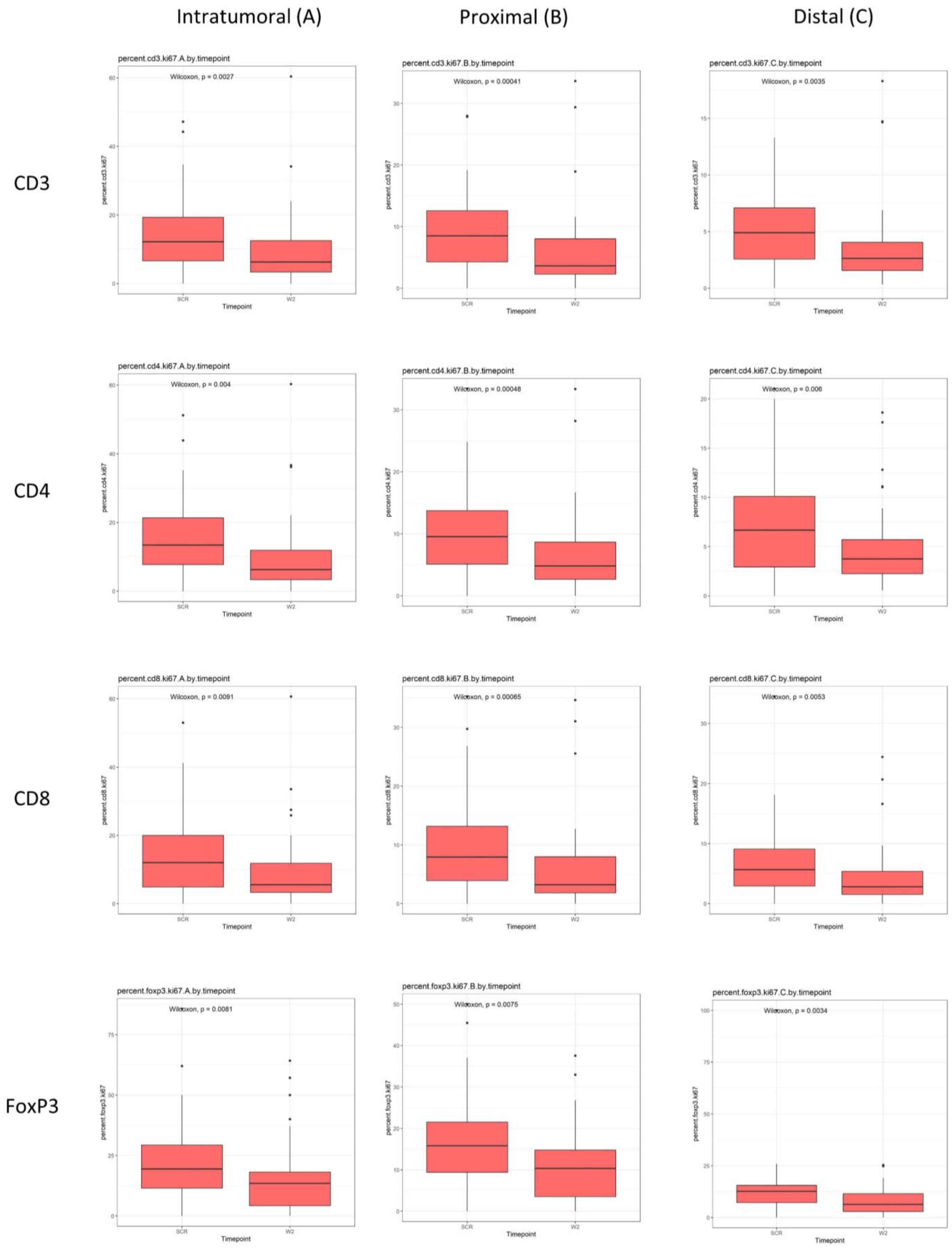

Supplementary Figure 18. Box-plots of percentage of proliferating immune cells (CD3+, CD4+, CD8+ and Foxp3+) by timepoint in intratumoral (A), proximal (B) and distal (C) locations. Boxplot legend: centre line: median; bounds of box: interquartile range (IQR); whiskers: highest and lowest value excluding outliers (Q3+1.5*IQR to Q1-1.5*IQR); markers beyond the whiskers: potential outliers.

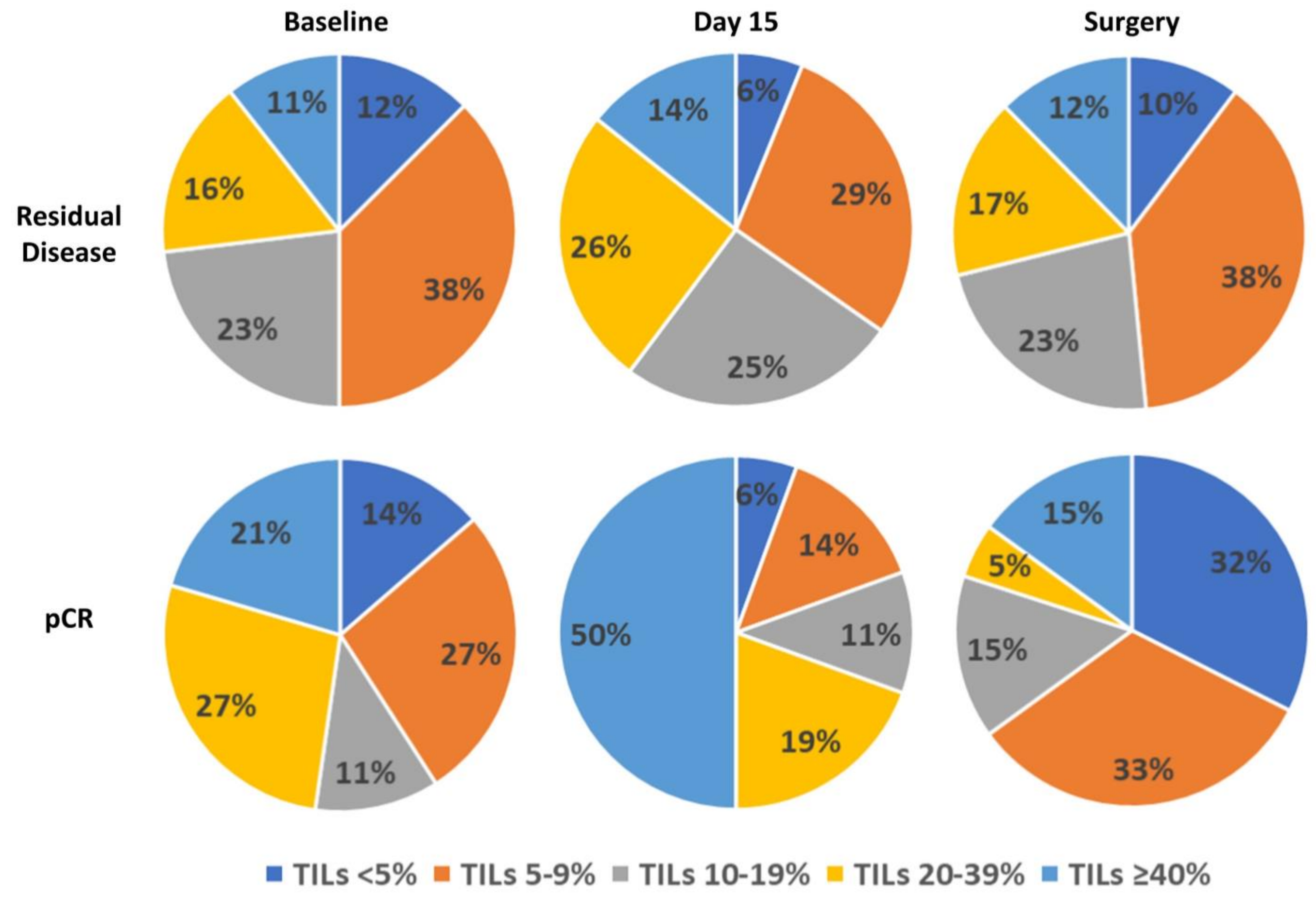

Supplementary Figure 19. Distribution of sTIL levels at the three timepoints according to pathological response (pCR versus residual disease).

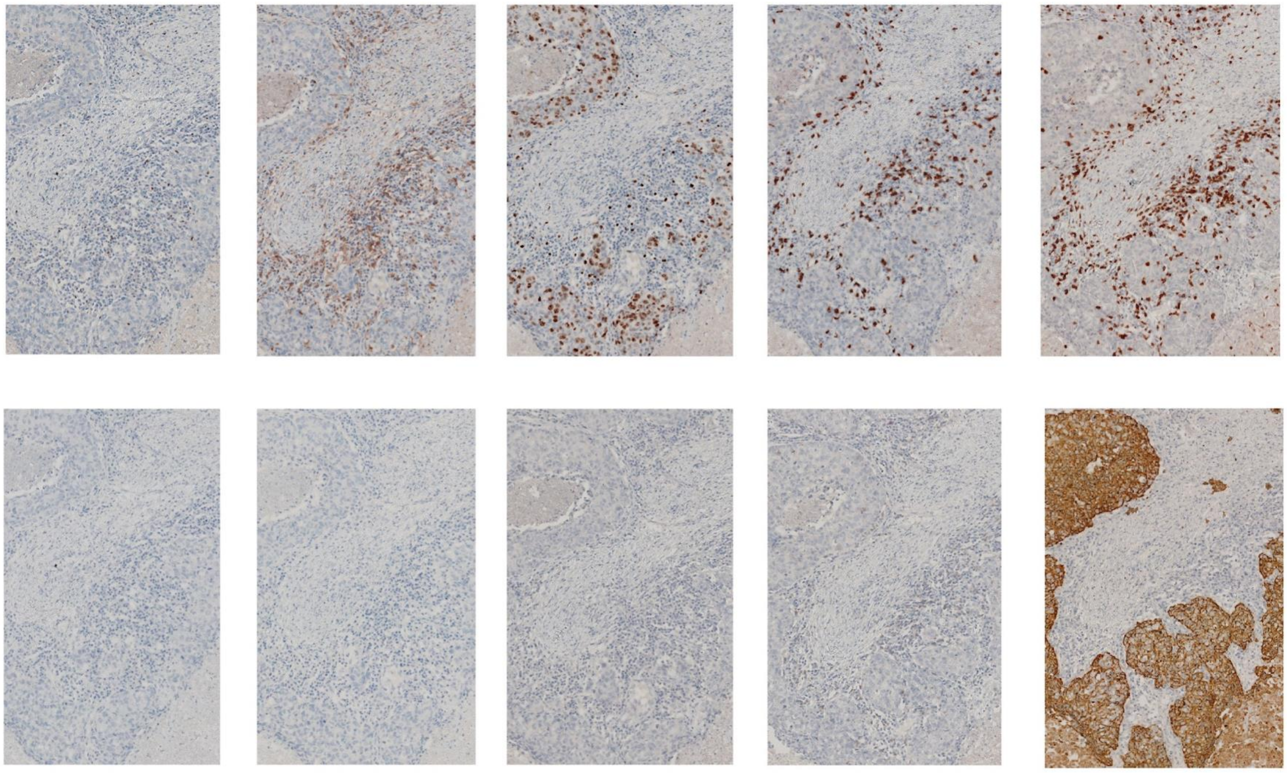

Supplementary Figure 20. Sequential staining of Foxp3, CD4, KI67, CD8 and CD3 on the same tissue slide (top panel, from left to right). In the bottom panel, an intermediate step in-between two target stainings where the primary antibody was omitted shows the absence of cross-reactivity. The last staining shows a cytokeratin after CD3 staining for tumor mask. Images at 4X.
