# Supplementary material for: Immune microenvironment characterisation and dynamics during anti-HER2-based neoadjuvant treatment in HER2-positive breast cancer
Source: NPJ Precis Oncol. 2021 Mar 19;5:23. doi: 10.1038/s41698-021-00163-6 (PMC7979716; doi:10.1038/s41698-021-00163-6)
Supplement: Supplementary file 2 — REPORTING SUMMARY [file 41698_2021_163_MOESM2_ESM.pdf]

nature research

Corresponding author(s): Aleix Prat
Paolo Nuciforo

Last updated by author(s): Feb 8, 2021

Reporting Summary

Nature Research wishes to improve the reproducibility of the work that we publish. This form provides structure for consistency and transparency in reporting. For further information on Nature Research policies, see our Editorial Policies and the Editorial Policy Checklist.

Statistics

For all statistical analyses, confirm that the following items are present in the figure legend, table legend, main text, or Methods section.

n/a	Confirmed	
☐	☒	The exact sample size (*n*) for each experimental group/condition, given as a discrete number and unit of measurement
☒	☐	A statement on whether measurements were taken from distinct samples or whether the same sample was measured repeatedly
☐	☒	The statistical test(s) used AND whether they are one- or two-sided<br>*Only common tests should be described solely by name; describe more complex techniques in the Methods section.*
☐	☒	A description of all covariates tested
☐	☒	A description of any assumptions or corrections, such as tests of normality and adjustment for multiple comparisons
☐	☒	A full description of the statistical parameters including central tendency (e.g. means) or other basic estimates (e.g. regression coefficient) AND variation (e.g. standard deviation) or associated estimates of uncertainty (e.g. confidence intervals)
☐	☒	For null hypothesis testing, the test statistic (e.g. *F*, *t*, *r*) with confidence intervals, effect sizes, degrees of freedom and *P* value noted<br>*Give P values as exact values whenever suitable.*
☒	☐	For Bayesian analysis, information on the choice of priors and Markov chain Monte Carlo settings
☒	☐	For hierarchical and complex designs, identification of the appropriate level for tests and full reporting of outcomes
☐	☒	Estimates of effect sizes (e.g. Cohen's *d*, Pearson's *r*), indicating how they were calculated

Our web collection on statistics for biologists contains articles on many of the points above.

Software and code

Policy information about availability of computer code

Data collection	All statistical analyses were performed using the R software 3.6.1.
Data analysis	All statistical analyses were performed using the R software 3.6.1.

For manuscripts utilizing custom algorithms or software that are central to the research but not yet described in published literature, software must be made available to editors and reviewers. We strongly encourage code deposition in a community repository (e.g. GitHub). See the Nature Research guidelines for submitting code & software for further information.

Data

Policy information about availability of data

All manuscripts must include a data availability statement. This statement should provide the following information, where applicable:

- Accession codes, unique identifiers, or web links for publicly available datasets
- A list of figures that have associated raw data
- A description of any restrictions on data availability

The data generated and analysed during this study are described in the following data record: https://doi.org/10.6084/m9.figshare.13681456 (24). The data files underlying the related study are available from the corresponding authors upon reasonable request. However, several files are not publicly available in order to protect patient privacy. A comprehensive list of data files underlying the related manuscript along with details of their availability is contained in the spreadsheet 'Griguolo_et_al_2021_underlying_datafile_list.xlsx', available as part of the figshare. The custom-developed algorithms (T-cell APP) created using the Author® module of VISIOPHARM® (VIS) Image Analysis Software (Visiopharm Integrator System version 2019.02.1.6005, Visiopharm, Denmark) are also available as part of the figshare data record.

Field-specific reporting

Please select the one below that is the best fit for your research. If you are not sure, read the appropriate sections before making your selection.

☒ Life sciences ☐ Behavioural & social sciences ☐ Ecological, evolutionary & environmental sciences

For a reference copy of the document with all sections, see nature.com/documents/nr-reporting-summary-flat.pdf

Life sciences study design

All studies must disclose on these points even when the disclosure is negative.

Sample size	Samples collected during phase II PAMELA trial and available for gene-expression analysis and for multiplex IHC analysis were assessed.
Data exclusions	Samples with total region of interest below 100000 µm were excluded from the multiplex IHC analysis.
Replication	No replications attempted (only few clinical trials tested chemo-free neoadjuvant regimens for HER2+ breast cancer and sample availability is limited).
Randomization	No randomization. Association of biological characteristics and response to treatment was assessed.
Blinding	No allocation by investigators to different treatment groups.

Reporting for specific materials, systems and methods

We require information from authors about some types of materials, experimental systems and methods used in many studies. Here, indicate whether each material, system or method listed is relevant to your study. If you are not sure if a list item applies to your research, read the appropriate section before selecting a response.

Materials & experimental systems

n/a	Involved in the study	
☐	☒	Antibodies
☒	☐	Eukaryotic cell lines
☒	☐	Palaeontology and archaeology
☒	☐	Animals and other organisms
☐	☒	Human research participants
☐	☒	Clinical data
☒	☐	Dual use research of concern

Methods

n/a	Involved in the study	
☒	☐	ChIP-seq
☒	☐	Flow cytometry
☒	☐	MRI-based neuroimaging

Antibodies

Antibodies used	Detailed in Supplementary Table S18 of the manuscript.
Validation	Validation of the antibodies used is extensively detailed in the method section of the manuscript. Validated protocols are detailed in Supplementary Table S18. Comparisons with regular IHC stainings are shown in Supplementary Figure S1 and correlations with regular IHC by image analysis are shown in Supplementary Figure S2

Human research participants

Policy information about studies involving human research participants

Population characteristics	Detailed in the previous manuscript reporting clinical data from the PAMELA trial (Llombart-Cussac A. et al. Lancet Oncol 2018 https://doi.org/10.1016/S1470-2045(17)30021-9)
Recruitment	Detailed in the previous manuscript reporting clinical data from the PAMELA trial (Llombart-Cussac A. et al. Lancet Oncol 2018 https://doi.org/10.1016/S1470-2045(17)30021-9)
Ethics oversight	The study protocol was approved by independent ethics committees at each center (trial centers listed at clinicaltrials.gov, NCT01973660).

Note that full information on the approval of the study protocol must also be provided in the manuscript.

Clinical data

Policy information about clinical studies

All manuscripts should comply with the ICMJE guidelines for publication of clinical research and a completed CONSORT checklist must be included with all submissions.

Clinical trial registration	NCT01973660
Study protocol	The study protocol defined the PAMELA clinical trial (previously reported in Llombart-Cussac A. et al. Lancet Oncol 2018 https://doi.org/10.1016/S1470-2045(17)30021-9)
Data collection	Detailed in the previous manuscript reporting clinical data from the PAMELA trial (Llombart-Cussac A. et al. Lancet Oncol 2018 https://doi.org/10.1016/S1470-2045(17)30021-9)
Outcomes	Detailed in the previous manuscript reporting clinical data from the PAMELA trial (Llombart-Cussac A. et al. Lancet Oncol 2018 https://doi.org/10.1016/S1470-2045(17)30021-9)
